# Differing impact of phosphoglycerate mutase 1-deficiency on brown and white adipose tissue

**DOI:** 10.1016/j.isci.2022.104268

**Published:** 2022-04-18

**Authors:** Yohko Yoshida, Ippei Shimizu, Yung-Ting Hsiao, Masayoshi Suda, Goro Katsuumi, Masahide Seki, Yutaka Suzuki, Shujiro Okuda, Tomoyoshi Soga, Tohru Minamino

**Affiliations:** 1 Department of Cardiovascular Biology and Medicine, Juntendo University Graduate School of Medicine, 2-1-1 Hongo, Bunkyo-ku, Tokyo 113-8421, Japan; 2 Department of Advanced Senotherapeutics, Juntendo University Graduate School of Medicine, Tokyo 113-8421, Japan; 3 Department of Computational Biology and Medical Sciences, Graduate School of Frontier Sciences, The University of Tokyo, Chiba 277-8561, Japan; 4 Division of Bioinformatics, Niigata University Graduate School of Medical and Dental Sciences, Niigata 951-8510, Japan; 5 Institute for Advanced Biosciences, Keio University, Yamagata 997-0052, Japan; 6 Japan Agency for Medical Research and Development-Core Research for Evolutionary Medical Science and Technology (AMED-CREST), Japan Agency for Medical Research and Development, Tokyo 100-0004, Japan

**Keywords:** Physiology, Molecular physiology, Molecular biology

## Abstract

Brown adipose tissue (BAT) is a metabolically active organ that contributes to the thermogenic response to cold exposure. In addition, other thermogenic cells termed beige adipocytes are generated in white adipose tissue (WAT) by cold exposure. Although activation of brown/beige adipose tissue is associated with mobilization of both glucose and lipids, few studies have focused on the role of glycolytic enzymes in regulating adipose tissue function. We generated mouse models with specific deletion of the glycolytic enzyme phosphoglycerate mutase 1 (PGAM1) from adipose tissue. Deletion of Pgam1 from both BAT and WAT promoted whitening of BAT with beiging of visceral WAT, whereas deletion of Pgam1 from BAT alone led to whitening of BAT without beiging of WAT. Our results demonstrate a potential role of glycolytic enzymes in beiging of visceral WAT and suggest that PGAM1 would be a novel therapeutic target in obesity and diabetes.

## Highlights

• Pgam1 deletion leads to whitening of brown adipose tissue
• Pgam1 deletion promotes beiging of visceral white adipose tissue (WAT)
• Pgam1 deletion-induced beiging is associated with increased levels of amino acids

## Graphical abstract

**Figure:**
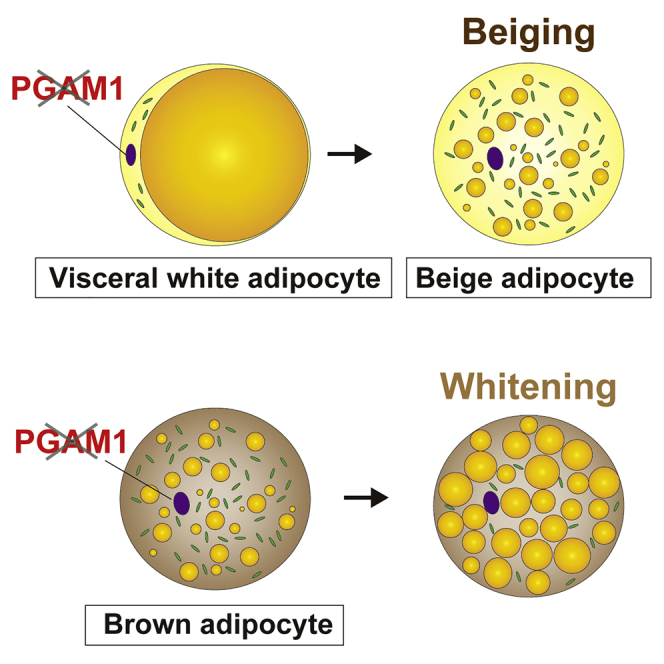

## Introduction

The number of patients with obesity or diabetes has increased dramatically in the modern world, and these conditions have become major health problems for many societies (Van Gaal et al., 2006; Yanovski and Yanovski, 2002). It has been suggested that a potential therapeutic strategy for diabetes and its complications could involve activation of brown adipose tissue (BAT), which normally consumes excessive calories by generating heat in response to cold exposure (Hankir et al., 2016). Cold stimulates the sympathetic nervous system to promote lipolysis and thermogenesis, and is believed to be the most potent activator of BAT. Several studies have demonstrated that chronic acclimation of adult humans to cold increases the amount of activated BAT in the supraclavicular region, and this change is associated with improvement of insulin sensitivity (Lee et al., 2014) or with an increase of cold-induced energy expenditure (van der Lans et al., 2013; Yoneshiro et al., 2013). However, activation of BAT is impaired by aging, obesity, and the diabetic state in humans and in animal models (Cypess et al., 2009; Rogers et al., 2012; Yoneshiro et al., 2011), although the underlying molecular mechanisms have not been fully elucidated.

Cold exposure also activates inducible thermogenic adipocytes in white adipose tissue (WAT). These cells, known as beige adipocytes, appear to possess specific regulatory machinery for a thermogenic response and have a distinct developmental origin from brown adipocytes (Harms and Seale, 2013; Wu et al., 2012). In mice, inguinal WAT (iWAT) is known to readily undergo “beiging” in response to cold exposure, whereas gonadal WAT (gWAT) shows a much weaker beiging response, presumably because of differential expression of transcription factors involved in brown/beige adipocyte differentiation (Kajimura et al., 2015). In humans, supraclavicular BAT has a similar molecular signature to that of mouse beige adipocytes (Lee et al., 2014; Sharp et al., 2012; Shinoda et al., 2015; Wu et al., 2012), whereas BAT at other sites expresses classical BAT markers (Cypess et al., 2013; Nagano et al., 2015; Xue et al., 2015). A number of research groups have identified factors that regulate the activity of BAT and/or beige adipose tissue and could be potential therapeutic targets (Reitman, 2017; Villarroya and Vidal-Puig, 2013). These molecules include a number of growth factors and cytokines, such as fibroblast growth factor 21 (Fisher et al., 2012), adiponectin (Hui et al., 2015), natriuretic peptides (Bordicchia et al., 2012), prostaglandins (Vegiopoulos et al., 2010), and irisin (Bostrom et al., 2012). Thyroid hormone and parathyroid hormone are also known as BAT activators (Kir et al., 2016; Lin et al., 2015). Moreover, various transcription factors (Ma et al., 2015; Tai et al., 1996) and signaling molecules (Bi et al., 2014; Whittle et al., 2012) were reported to be involved in activating BAT and beige adipose tissue. Although β_3_-adrenergic stimulation is critical for activating thermogenic modulation by BAT and beige adipose tissue, other neurotransmitters such as adenosine (Gnad et al., 2014), serotonin (Crane et al., 2015), gamma-aminobutyric acid (Ikegami et al., 2018) also make a contribution.

Activation of BAT is associated with increased mobilization of glucose and lipids, which are utilized for generation of heat, and therefore BAT acts as a metabolic sink for these energy sources (Bartelt et al., 2011; Seale et al., 2011). The interaction between lipid metabolism and activation of BAT has been extensively investigated. For example, studies involving genetic deletion of factors that promote fatty acid oxidation have revealed an essential role of these factors in BAT activation by cold exposure (Ji et al., 2008; Lee et al., 2015; Tolwani et al., 2005). Inhibition of intracellular triglyceride lipolysis suppresses cold-induced activation of BAT in humans and animals, but lipolytic activity in BAT itself is not essential because defective BAT lipolysis can be fully compensated by circulating fatty acids derived from WAT (Schreiber et al., 2017; Shin et al., 2017). It has also been reported that lipid biosynthesis is required for the thermogenic response of BAT because it modulates the expression of mitochondrial electron transport chain components (Tan et al., 2015). Accumulating evidence suggests a critical role of glycolysis in regulating adipose tissue function (Hankir and Klingenspor, 2018; Isidor et al., 2020; Jung et al., 2021; Nguyen et al., 2020), but it remains unclear how glycolytic enzymes differently act on various adipose tissues. Therefore, we investigated whether BAT and WAT were regulated by the glycolytic enzyme phosphoglycerate mutase (PGAM), which catalyzes conversion of 3-phosphoglycerate (3PG) to 2-phosphoglycerate (2PG), by generating mice with adipose tissue-specific PGAM deficiency.

## Results

### Adipose tissue-specific Pgam1 deletion has differing effects on BAT and WAT

**Figure 1:**
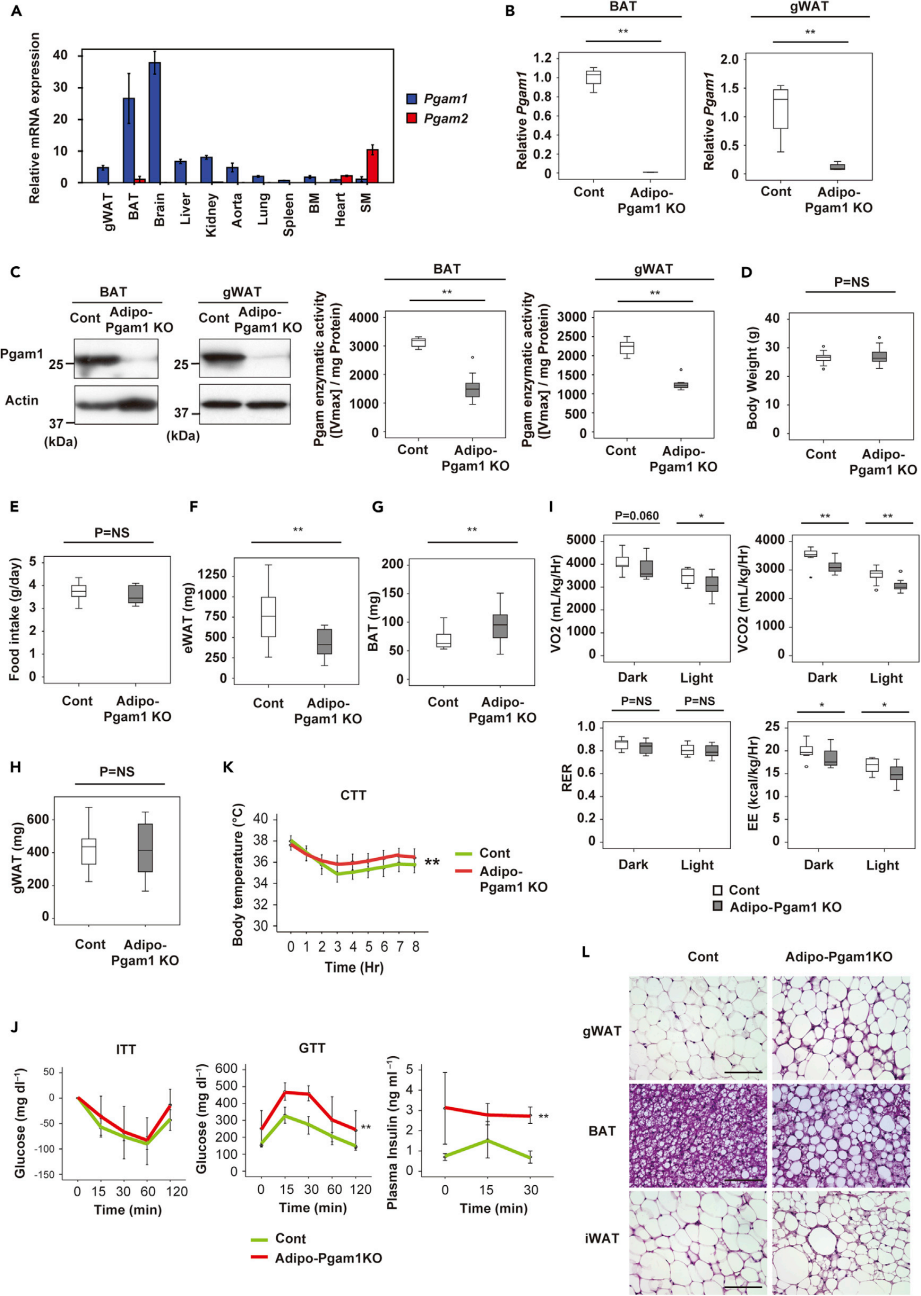
Adipose tissue-specific Pgam1 deletion has differing effects on BAT and WAT (A) Relative transcription level for *Pgam1* and *Pgam2* of whole body organs from 12-weeks-old C57BL/6N mice (each n = 4). (B) Transcripts for *Pgam1* of BAT (left panel) and gonadal WAT (gWAT) (right panel) from Adipo-Pgam1 knockout (Adipo-Pgam1 KO) and their littermate WT (Cont) mice (BAT; n = 4, 4, gWAT; n = 4, 4). (C) Left; Western blot analysis for Pgam1 in BAT (left) and gWAT (right) prepared in (B). Actin was used as loading control. Right; Pgam enzymatic activity of BAT (left) and gWAT (right) prepared in (B) (BAT; n = 9, 9, gWAT; n = 9, 9). (D) Body weight of mice prepared in (B) (n = 19, 16). (E) Food intake of mice prepared in (B) (n = 11, 8). (F) Visceral fat weight of mice prepared in (B) analyzed with CT scan (n = 12, 9). (G) BAT weight of mice prepared in (B) (n = 19, 16). (H) gWAT weight of mice prepared in (B) (n = 19, 16). (I) Oxygen consumption (VO_2_)(n = 16, 10), CO_2_ emission (VCO_2_)(n = 16, 10), respiratory exchange ratio (RER)(n = 16, 10) and energy expenditure (EE)(n = 16, 10) of mice prepared in (B). (J) Insulin tolerance test (ITT) (left) (n = 9, 4), glucose tolerance test (GTT) (middle) (n = 9, 4) and plasma insulin concentration during GTT (right) (n = 7, 3) of mice prepared in (B). (K) Acute cold tolerance test in mice prepared in mice prepared in (B) (n = 16, 13). (L) Hematoxylin and eosin (HE) staining of gWAT (upper panel), BAT (middle panel) and iWAT (lower panel) from mice prepared in (B). Scale bar = 100μm. Data were analyzed by the 2-tailed Student’s *t* test (B–I), or repeated measures followed by Tukey’s multiple comparison test (J and K). ∗p < 0.05, ∗∗p < 0.01. Values represent the mean ± SEM NS = not significant. See also Figure S1.

We first determined the level of Pgam expression in various mouse tissues. PCR revealed high levels of Pgam1 expression in the brain and BAT, as well as moderate Pgam1 expression in the WAT, aorta, liver, and kidney (Figure 1A). Pgam2, a muscle-specific isoform, was predominantly expressed by cardiac and skeletal muscle (Figure 1A). Then we generated adipose tissue-specific Pgam1 knockout mice (Adipoq-Cre; floxed Pgam1, Adipo-Pgam1 KO mice). PCR showed that Pgam1 expression was significantly reduced in the BAT of these mice, as well as in gonadal WAT (gWAT) and inguinal WAT (iWAT) (Figures 1B and S1A). Western blot analysis also demonstrated significant downregulation of Pgam1 expression in BAT and WAT, along with reduced enzyme activity (Figure 1C). Both body weight and food intake showed no differences between Adipo-Pgam1 KO mice and their littermate controls (Figures 1D and 1E). Analysis of CT data revealed a decrease of total WAT weight in Adipo-Pgam1 KO mice (Figure 1F). In these mice, BAT weight was increased and iWAT weight was reduced, whereas gWAT weight was unchanged (Figures 1G, 1H, and S1B). Investigation of the metabolic profile of Adipo-Pgam1 KO mice demonstrated lower energy expenditure (Figure 1I) and glucose intolerance/hyperinsulinemia compared with their littermate controls (Figure 1J). Despite these metabolic abnormalities, Adipo-Pgam1 KO mice showed superior cold tolerance compared with littermate controls (Figure 1K). Histological examination of Adipo-Pgam1 KO mice showed that the BAT of these animals contained larger lipid droplets and had undergone whitening, whereas gWAT and iWAT demonstrated an increase of beige adipocytes (Figure 1L). We also found an increase of macrophage infiltration into WAT of Adipo-Pgam1 KO mice (Figures S1C and S1D). These findings suggested that BAT dysfunction and WAT inflammation induced by Pgam1 deficiency led to abnormal glucose metabolism, but beiging of WAT contributed to preservation of the thermogenic response.

### Opposing metabolic profiles of BAT and WAT in Adipo-Pgam1 KO mice

**Figure 2:**
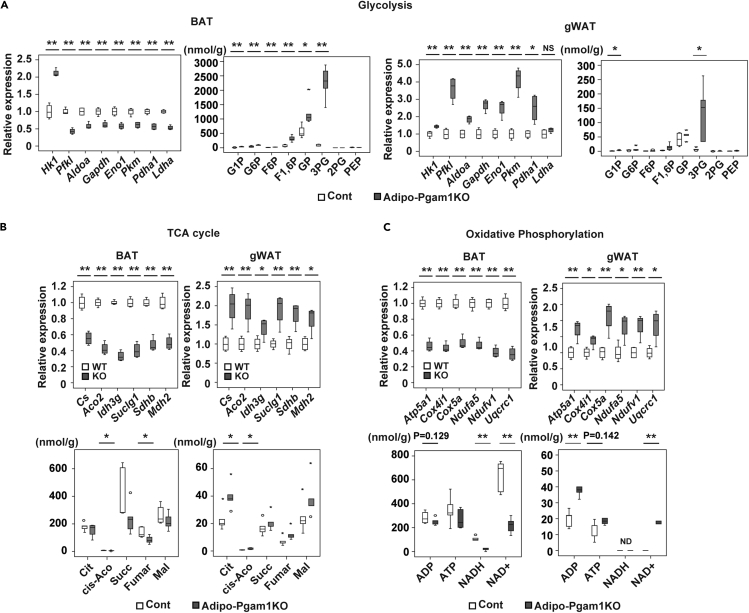
Opposing metabolic profiles of BAT and WAT in Adipo-Pgam1 KO mice (A) Relative change of transcripts (each n = 4, 4) and the tissue weight-adjusted metabolite level (each n = 5, 5) in glycolytic pathway in BAT and gWAT from Adipo-Pgam1 KO mice. (B) Relative change of transcripts (each n = 4, 4) and the tissue weight-adjusted metabolite level (each n = 5, 5) in TCA cycle in BAT (left) and gWAT (right) from Adipo-Pgam1 KO mice. (C) Relative change of transcripts (each n = 4, 4) and the tissue weight-adjusted metabolite level (each n = 5, 5) in oxidative phosphorylation in BAT and gWAT from Adipo-Pgam1 KO mice. Data were analyzed by the 2-tailed Student’s *t* test (A, B and C). ∗p < 0.05, ∗∗p < 0.01. Values represent the mean ± SEM NS = not significant. See also Figures S2 and S3.

To investigate the mechanisms underlying the phenotypic changes of BAT and WAT induced by Pgam1 deficiency, we conducted transcriptomic and metabolomic analyses of Adipo-Pgam1 KO mice and littermate controls. Transcriptomic analysis demonstrated marked downregulation of enzymes involved in glycolysis and the tricarboxylic acid (TCA) cycle in BAT from Adipo-Pgam1 KO mice compared with BAT from littermate controls (Figures 2A and 2B), corresponding to the observed whitening of BAT in the KO mice (Figure 1L). In contrast, expression of these enzymes was upregulated in WAT from Adipo-Pgam1 KO mice, particularly gWAT, compared with WAT from littermate controls (Figures 2A, 2B, S2A, and S2B). Metabolomic analysis of the glycolytic pathway revealed a marked increase of 3PG in BAT and WAT confirming *Pgam1* gene deletion (Figures 2A and S2B). Consistent with the results of transcriptomic analysis of the TCA cycle, intermediate metabolites of this cycle were decreased in BAT and increased in WAT from Adipo-Pgam1 KO mice compared with the corresponding tissues from littermate controls (Figures 2B and S2B). Similarly, components of the mitochondrial oxidative phosphorylation system showed downregulation in BAT versus upregulation in WAT from Adipo-Pgam1 KO mice (Figures 2C, S2A, and S2B). Gene ontology enrichment analysis indicated the development of an inflammatory response and metabolic dysfunction in BAT from Adipo-Pgam1 KO mice (Figure S3). In contrast, WAT from these mice showed enhancement of metabolic activity, supporting the observed phenotypic changes of adipose tissue (Figure S3).

### Adipo-Pgam1 KO mice show whitening of BAT and beiging of WAT

**Figure 3:**
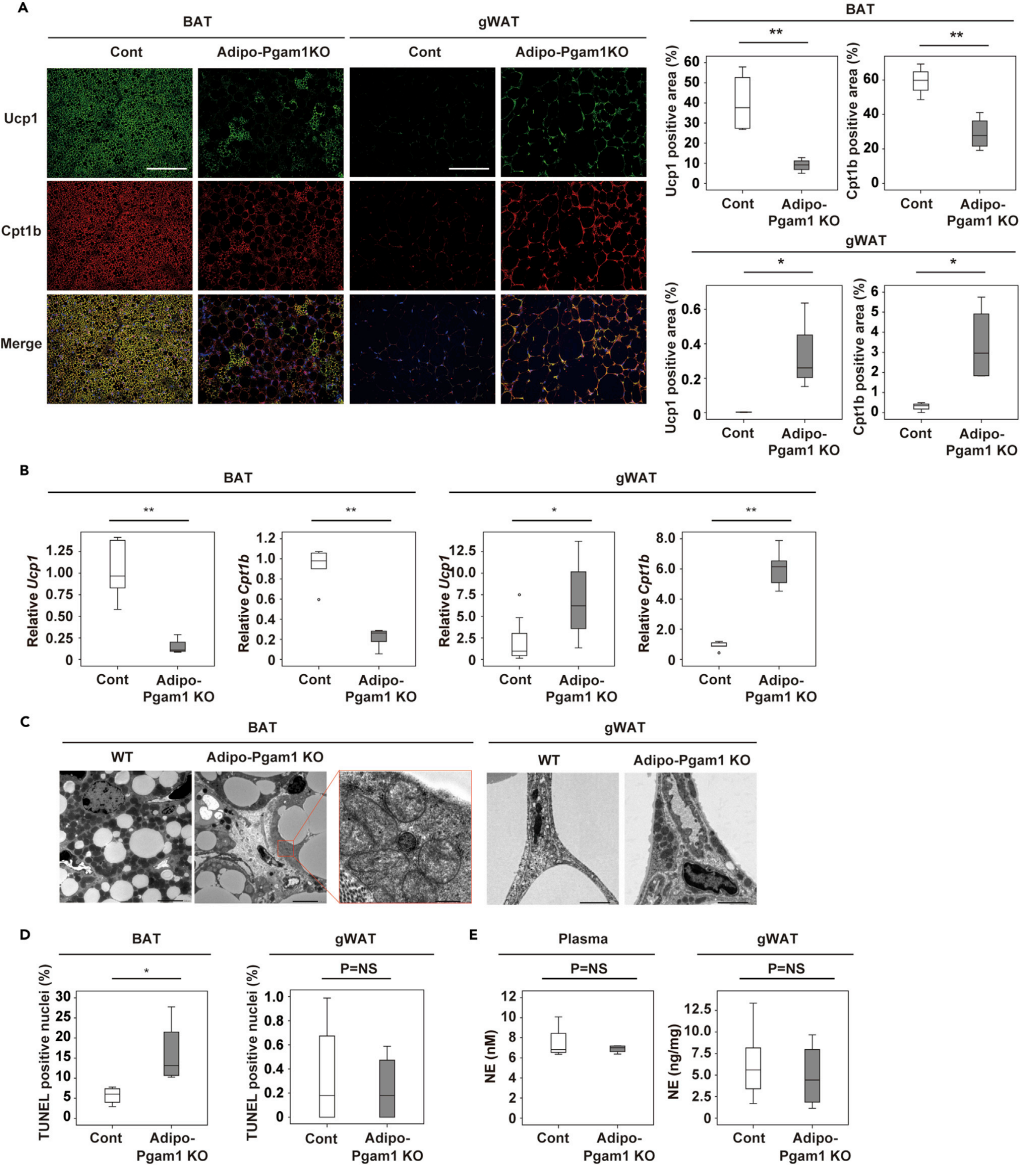
Adipo-Pgam1 KO mice show whitening of BAT and beiging of WAT (A) Immunofluorescent staining showing Ucp1 (green) and Cpt1b (red) in BAT (left) and gWAT (right) from Adipo-Pgam1 KO and their littermate WT mice. Scale bar = 100 μm. The graphs on the right display the Ucp1-or Cpt1b-positive area (%) (each n = 4, 4). (B) Transcripts for *Ucp1* and *Cpt1b* of BAT (left) and gWAT (right) from mice prepared in (A) (BAT; *Ucp1* n = 8, 4, *Cpt1b* n = 6, 5, gWAT; *Ucp1* n = 8, 4, *Cpt1b* n = 6, 5). (C) Transmission electron microscopy analyzing BAT (left) and gWAT (right) from mice prepared in (A). Scale bar = 5μm for low magnification and 500 nm for high magnification in BAT and 2μm in gWAT. (D) Quantification of TUNEL positive cells of BAT (left) and gWAT (right) from mice prepared in (A) (n = 4,4). (E) Norepinephrine level in the circulation (left) and gWAT (right) from mice prepared in (A). (Plasma; n = 9, 4, gWAT; n = 6, 4). Data were analyzed by the 2-tailed Student’s *t* test (A, B, D, and E). ∗p < 0.05, ∗∗p < 0.01. Values represent the mean ± SEM NS = not significant. See also Figures S4–S6.

To further investigate the adipose tissues changes in Adipo-Pgam1 KO mice, we performed histological examination by using uncoupled protein 1 (Ucp1) and carnitine palmitoyl transferase 1b (Cpt1b) as markers for thermogenic adipocytes. Compared with their littermate controls, the number of Ucp1-positive cells was significantly decreased in BAT from Adipo-Pgam1 KO mice, whereas it was significantly increased in gWAT from these mice (Figure 3A). Likewise, there were fewer Cpt1b-positive cells in BAT from Adipo-Pgam1 KO mice and more Cpt1b-positive cells in gWAT from these mice than in the corresponding tissues from littermate controls (Figure 3A). PCR revealed upregulated expression of Ucp1 and Cpt1b in gWAT from Adipo-Pgam1 KO mice, whereas expression was downregulated in BAT (Figure 3B). Similar results were observed in expression of other beige makers (Figure S4). It is interesting that iWAT showed milder beiging than gWAT in these mice after Pgam1 deletion, including a smaller increase of Ucp1 or Cpt1b-positive cells and less marked upregulation of beige adipocyte markers (Figures S5A–S5C). In Adipo-Pgam1 KO mice, electron microscopy detected a number of mitochondria with structural collapse in BAT, whereas there was an increase of normal mitochondria in gWAT (Figure 3C) and also in iWAT to a lesser extent (Figure S5D). We also observed a significant increase of TUNEL-positive cells in BAT from Adipo-Pgam1 KO mice (Figure 3D). Norepinephrine was not elevated in plasma or in WAT from Adipo-Pgam1 KO mice (Figure 3E), suggesting that beiging of gWAT after Pgam1 deletion was not mediated by neural activation because of BAT dysfunction.

### BAT-specific Pgam1 deletion does not promote beiging of WAT

**Figure 4:**
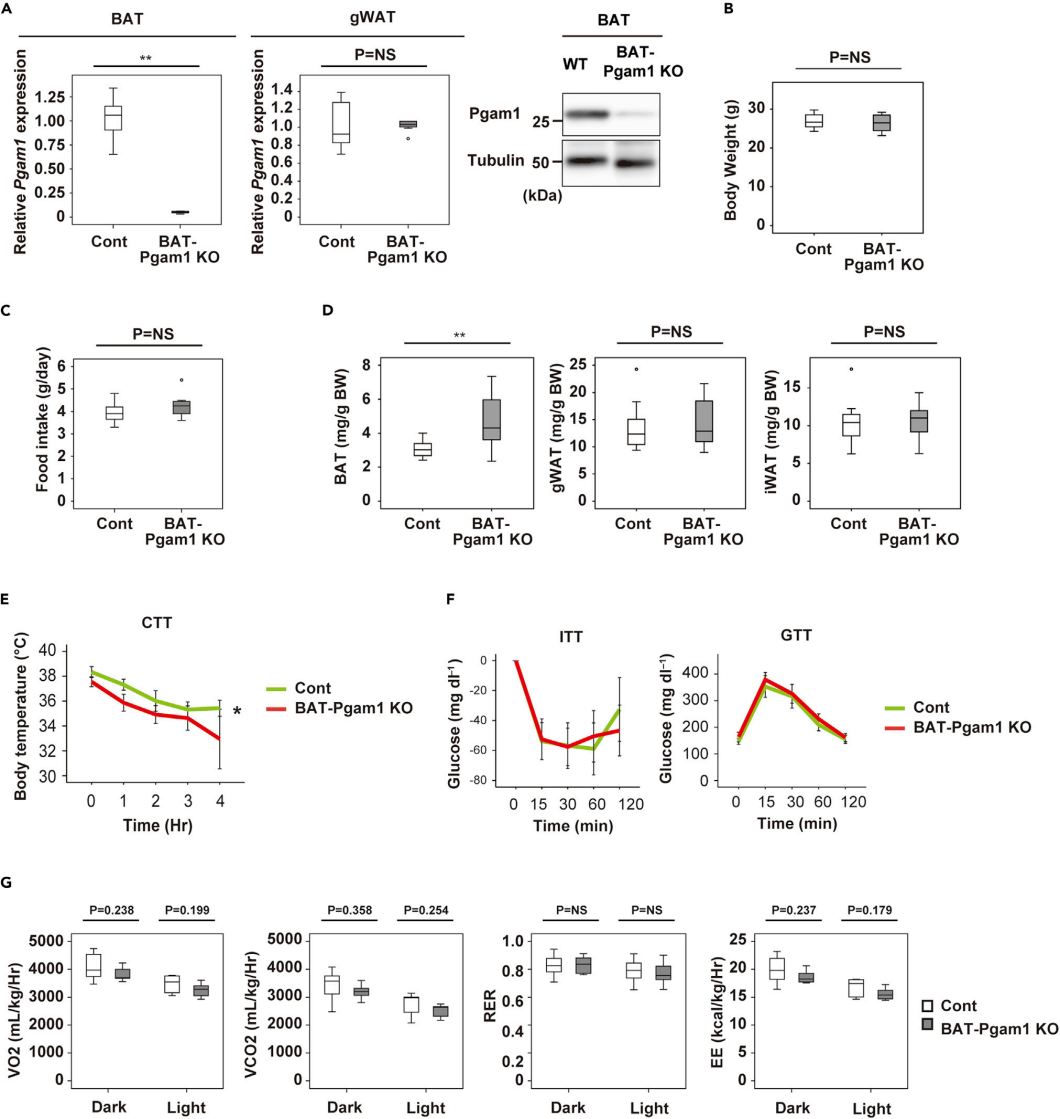
Metabolic phenotypes of BAT-specific Pgam1 KO mice (A) Transcripts for *Pgam1* of BAT (left graph) and gWAT (right graph) from BAT-Pgam1 knockout (BAT-Pgam1 KO) and their littermate WT (Cont) mice (BAT; n = 7, 7, gWAT; n = 7, 7). Western blot analysis showing down-regulation of Pgam1 in BAT from BAT-Pgam1 KO mice. (B) Body weight of mice prepared in (A) (n = 12, 12). (C) Food intake of mice prepared in (A) (n = 7, 7). (D) Body weight-adjusted BAT (n = 12, 12), gWAT (n = 11, 12) and inguinal WAT (iWAT) (n = 11, 12) weight of mice prepared in (A). (E) Acute cold tolerance test in mice prepared in mice prepared in (A) (n = 12, 12). (F) Insulin tolerance test (ITT) (left) (n = 12, 13) and glucose tolerance test (GTT) (right) (n = 12, 13) of mice prepared in (A). (G) Oxygen consumption (VO_2_)(n = 7, 7), CO_2_ emission (VCO_2_)(n = 7, 7), respiratory exchange ratio (RER)(n = 7, 7) and energy expenditure (EE)(n = 7, 7) of mice prepared in (A). Data were analyzed by the 2-tailed Student’s *t* test (A–D and G), or repeated measures followed by Tukey’s multiple comparison test (E and F). ∗p < 0.05, ∗∗p < 0.01. Values represent the mean ± SEM NS = not significant.

**Figure 5:**
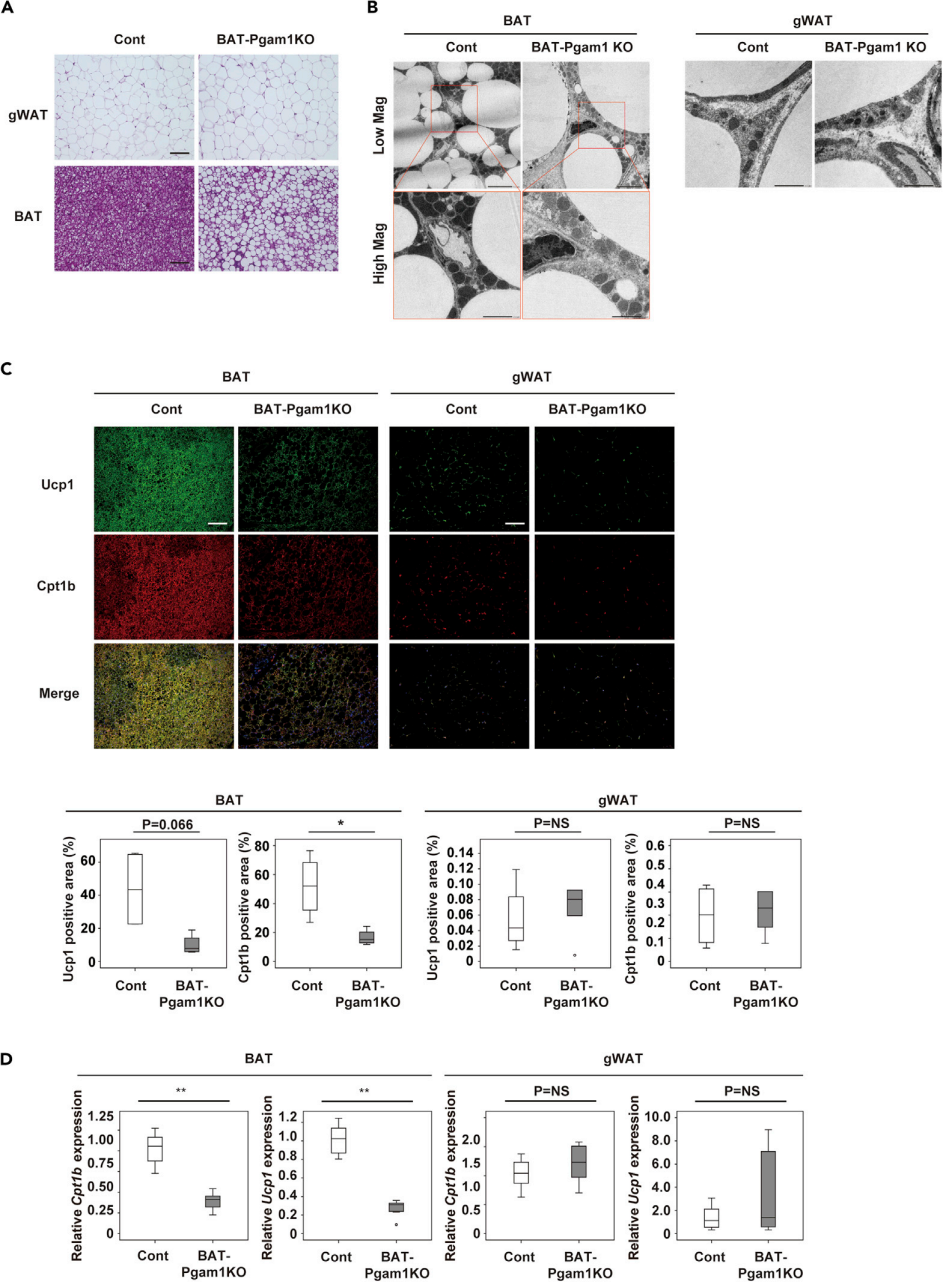
BAT-specific Pgam1 deletion does not promote beiging of WAT (A) Hematoxylin and eosin (HE) staining of gWAT (upper panel) and BAT (lower panel) from BAT-Pgam1 knockout (BAT-Pgam1 KO) and their littermate WT (Cont) mice. Scale bar = 100μm. (B) Transmission electron microscopy analyzing BAT (left) and gWAT (right) from mice prepared in (A). Scale bar = 5μm for low magnification and 2μm for high magnification in BAT and 2μm in gWAT. (C) Immunofluorescent staining showing Ucp1 (green) and Cpt1b (red) in BAT (left) and gWAT (right) from mice prepared in (A). Scale bar = 100 μm. The lower graphs display the Ucp1-or Cpt1b-positive area (%) (each n = 4, 4). (D) Transcripts for *Ucp1* and *Cpt1b* of BAT (left) and gWAT (right) from mice prepared in (A) (each n = 7, 7). Data were analyzed by the 2-tailed Student’s *t* test (C and D). ∗p < 0.05, ∗∗p < 0.01. Values represent the mean ± SEM NS = not significant.

It is possible that dysfunctional BAT promotes beiging of WAT via the central nervous system. To further investigate the mechanisms underlying Pgam1 deletion-induced beiging of gWAT, we generated BAT-specific Pgam1 knockout mice (Ucp1-Cre; floxed Pgam1, BAT-Pgam1 KO mice). In these mice, Pgam1 expression was significantly reduced in BAT, but not in WAT (Figure 4A). Body weight and food intake did not differ between BAT-Pgam1 KO mice and their littermate controls (Figures 4B and 4C). BAT-Pgam1 KO mice showed a significant increase of BAT weight, whereas there were no changes of gWAT or iWAT weights (Figure 4D). Targeted deletion of Pgam1 from BAT led to impairment of cold tolerance with normal glucose and insulin tolerance (Figures 4E and 4F). We found that BAT-Pgam1 KO mice demonstrated lower energy expenditure compared with their littermate controls (Figure 4G), although the difference was not statistically significant. Histological examination revealed that deletion of Pgam1 from BAT led to whitening of BAT, but did not affect WAT (Figure 5A). In comparison with their littermate controls, electron microscopy showed that Pgam1 deletion was associated with an increase of abnormal mitochondria in BAT from Adipo-Pgam1 KO mice, but no specific changes were observed in WAT (Figure 5B). Although Ucp1-positive cells and Cpt1b-positive cells were decreased in BAT from BAT-Pgam1 KO mice, these cells were not increased in WAT (Figure 5C). According to PCR analysis, there was downregulation of thermogenic adipocyte markers in BAT from BAT-Pgam1 KO mice compared with littermate controls, whereas there was no difference of these markers in WAT (Figure 5D). Taken together, these findings suggested that Pgam1 deletion directly induces beiging of gWAT.

To test this hypothesis, we crossed Adipo-Pgam1 KO mice with EGFP Cre reporter mice (Adipoq-Cre; floxed Pgam1; CAG-LSL-EGFP). In these mice, expression of EGFP and Cpt1b was co-localized in gWAT (Figure S6A). We also found that Cre protein immunoreactivity was co-localized with that of Cpt1b in gWAT (Figure S6B), suggesting specific upregulation of Cpt1b expression in gWAT cells with Pgam1 deletion. To further examine whether Pgam1 deletion induces beiging of gWAT, we conducted single cell RNA sequence analysis of the stromal vascular fraction from gWAT of Adipo-Pgam1 KO mice and their littermate control mice. We identified a cluster in the fibroblast cluster of gWAT of littermate control mice that was rich for adipocyte markers (Fibroblast4; Figure S6C), suggesting that this population were preadipocytes and that Pgam1 was deleted in this population of Adipo-Pgam1 KO mice by the Cre recombination driven by the Adiponectin promoter. Consistent with this idea, the corresponding cell population in Adipo-Pgam1 KO mice showed a marked downregulation of Pgam1 expression. Analysis of the differential expressed genes in this cluster between genotypes revealed upregulation of genes indicative of beige cells (Figure S6D), suggesting a causative relationship between Pgam1 deletion and beiging of gWAT.

### Inhibition of glycolysis increases beige-like cells in adipocytes

**Figure 6:**
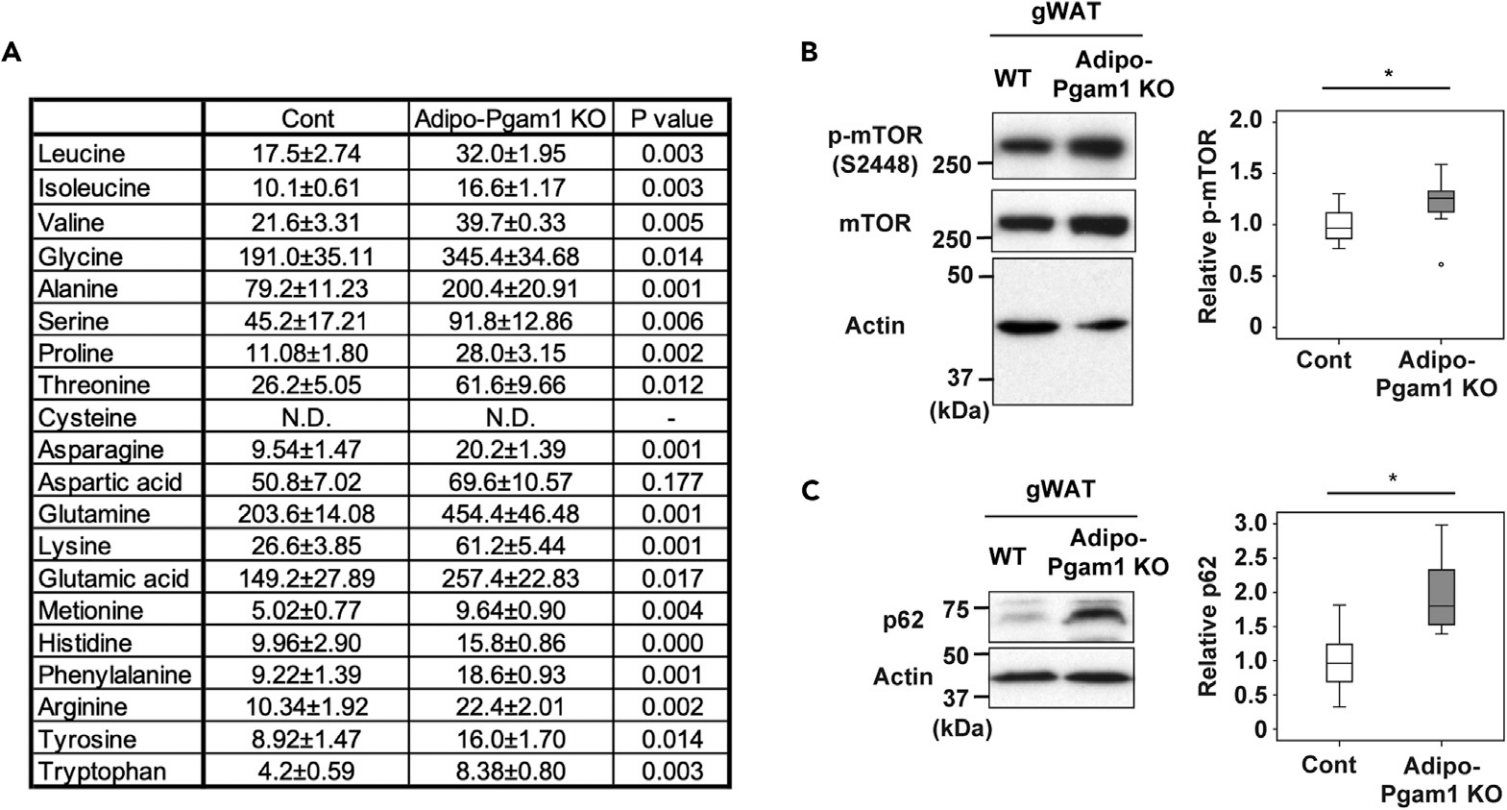
Pgam1 deletion affects BCAA metabolism (A) Tissue weight-adjusted amino acid levels (nmol/g) in gWAT from Adipo-Pgam1 KO and their littermate WT mice (n = 5, 5). (B) Western blot analysis for mTOR in gWAT prepared in (A). Actin was used as loading control. Right graphs show the quantification of *p*-mTOR/Actin ratio (n = 7,7). (C) Western blot analysis for p62 in gWAT prepared in (A). Actin was used as loading control. Right graphs show the quantification of p62 expression (n = 7,6). Data were analyzed by the 2-tailed Student’s *t* test (A–C). ∗p < 0.05, ∗∗p < 0.01. Values represent the mean ± SEM NS = not significant. See also Figures S7 and S8.

Gene set enrichment analysis indicated that there was marked downregulation of the degradation of branched amino acids (BCAA) in gWAT from Adipo-Pgam1 KO mice (Figure S3). In agreement with this finding, metabolomic analysis showed accumulation of various amino acids including BCAA in gWAT from these mice (Figure 6A). It is well accepted that an increase of branched amino acids leads to activation of the mTor pathway and inhibition of autophagy (Nie et al., 2018). We previously reported that dietary obesity promotes the whitening of BAT by activating mitophagy (Shimizu et al., 2014). In addition, Altshuler-Keylin et al. demonstrated that autophagy-mediated clearance of mitochondria is required for beige-to-white adipocyte phenotypic reversal and that inhibition of autophagy leads to maintenance of functioning beige adipocytes (Altshuler-Keylin et al., 2016). In agreement with our metabolomic data, in the present study we found upregulation of the mTor pathway in gWAT from Adipo-Pgam1 KO mice (Figure 6B); this upregulation was associated with increased expression of p62 (Figure 6C). We also examined BAT tissues and plasma from Adipo-Pgam1 KO and/or BAT-Pgam1 KO mice and found no significant changes in BCAA-mTor-autophagy signals (Figures S7A–S7E).

To examine a relationship between Pgam1 deletion and mTor-induced beiging in differentiated adipocytes, we infected NIH3T3L1 cells with an AAV vector that codes short hairpin (sh)-Pgam1. Consistent with the *in vivo* data from Adipo-Pgam1 KO mice, deletion of Pgam1 from differentiated adipocytes led to an increase in BCAA levels and activation of mTor signaling (Figures S8A–S8C). Pgam1 deletion upregulated expression of Ucp1 (Figure S8D), and this upregulation was attenuated by rapamycin (Figure S8E). Treatment of differentiated adipocytes with BCAA led to upregulation of Ucp1 (Figure S8F). Likewise, treatment of differentiated adipocytes with MHY1485, a potent mTOR activator that also inhibits autophagy, increased expression of p62 and Ucp1 (Figures S8G and S8H).

## Discussion

The present study demonstrated that deletion of Pgam1 from BAT and WAT had a different impact on each tissue, because it promoted whitening of BAT and beiging of WAT without any change of adipose tissue catecholamine levels. Deletion of Pgam1 from BAT alone did not affect WAT, excluding the possibility that BAT dysfunction induced beiging of WAT via activation of the sympathetic nervous system. These findings were further supported by *in vitro* experiments using differentiated adipocytes, which showed that deletion of Pgam1 led to an increase of beige cell makers, as well as by histological analysis of EGFP reporter mice. Taken together, our results indicate that beiging of WAT is induced by deletion of Pgam1 via a cell-autonomous mechanism.

It has been reported that Pgam1 has a role in tumorigenesis and cellular aging, and Pgam1 has been suggested as a therapeutic target for age-associated diseases including cancer (Hitosugi et al., 2012; Jiang et al., 2014; Kondoh et al., 2005). Interestingly, we observed upregulation of beige makers and downregulation of glycolytic enzymes in gWAT of aged mice with BAT whitening (data not shown), suggesting that inactivation of glycolysis may be involved in regulating the adaptive response of gWAT to age-associated BAT dysfunction. This adaptive response may provide a survival advantage for aged animals by maintaining minimal thermogenesis in response to cold and preventing inappropriate energy consumption under starvation conditions despite age-associated whitening of BAT. It is known that cold exposure preferentially promotes the beiging of iWAT rather than gWAT, whereas we found that Pgam1 deletion particularly increased beige-like cells in gWAT. Because visceral fat has a pathological role in metabolic dysfunction leading to type 2 diabetes, inhibition of glycolysis in gWAT could be an attractive strategy for treating metabolic diseases like type 2 diabetes.

In gWAT from Adipo-Pgam1 KO mice, we found accumulation of branched chain amino acids along with upregulation of the synthetic enzymes. Branched chain amino acids are the most abundant essential amino acids, and are not only substrates for the synthesis of nitrogenous compounds, but also act as signaling molecules in the regulation of glucose, lipid, and protein metabolism or immunity via special signaling networks such as the mTor pathway (Nie et al., 2018). Consistent with these reports, we identified activation of the mTor pathway in gWAT from Adipo-Pgam1 KO mice, which was associated with inhibition of autophagy and an increase of beige-like cells. Our transcriptomic analysis also demonstrated upregulation of the phosphoinositide 3-kinase/Akt pathway in gWAT from Adipo-Pgam1 KO mice that could possibly account for activation of mTor signaling (the Gene Expression Omnibus database GSE186030). When Halama et al. (2016) investigated the crucial metabolic pathways for differentiation of white adipocytes, their transcriptomic and metabolomic analyses identified phosphatidylcholine synthesis, fatty acids metabolism, and catabolism of branched chain amino acids as key regulatory pathways. They also demonstrated that a metabolic switch from catabolism of branched chain amino acids to lipid synthesis was crucial for white adipocyte differentiation. Taken together with our results, these findings suggest that branched chain amino acids may have a role in the regulation of white and beige adipocyte differentiation.

Our *in vitro* experiments using differentiated adipocytes suggested that autophagy could be involved in regulating the beiging of white adipocytes, but it remains to be determined whether Pgam1 deletion actually inhibits autophagy to promote the beiging response of gWAT. It would be interesting to examine the effect of specific mTor pathway activation (for example, using floxed Tsc1/2 mice) in gWAT of Adipo-Pgam1 KO mice, although a visceral WAT-specific Cre driver mouse is not available at present. Indeed, we still do not fully understand how Pgam1 deletion activates the mTor pathway, how Pgam1 deletion promotes accumulation of branched chain amino acids, or whether accumulation of these amino acids underlies activation of the mTor pathway and beiging of gWAT in Adipo-Pgam1 KO mice. Thus, further studies will be required to develop novel therapeutic strategies for metabolic disease based on modulation of cell metabolism in adipose tissue.

### Limitations of the study

The results of the present study suggest that the BCAA-mTor-autophagy axis may be involved in beiging of gWAT of Adipose-Pgam1 KO mice, but a causative relationship remains to be determined. Other factors, including other metabolites and insulin, that could potentially activate mTor signals may contribute to beiging of gWAT in Adipose-Pgam1 KO mice. Our study did not elucidate the mechanisms underlying upregulation of amino acids including BCAA in gWAT of Adipo-Pgam1 KO mice. We have not confirmed the changes in glycolysis and TCA in our mouse models by using flux analyses such as *in vivo* isotope tracing methods (Jung et al., 2021). We have not fully elucidated the mechanisms by which Adipo-Pgam1 KO mice show glucose intolerance along with normal thermogenic response.

## STAR★Methods

### Key resources table

**Table:** 

REAGENT or RESOURCE	SOURCE	IDENTIFIER
**Antibodies**		

anti-Pgam1 antibody	Abcam	ab2220, RRID: AB_302900
anti-β actin antibody(13 × 10^5^)	Cell Signaling	#4970, RRID: AB_2223172
anti-GAPDH antibody	Cell Signaling	#2118, RRID: AB_561053
anti-Tubulin antibody	Cell Signaling	#2125, RRID: AB_2619646
anti-p62 antibody	Abcam	ab219581, RRID: AB_219581
anti-phospho mTOR antibody	Cell Signaling	#2971, RRID: AB_330970
anti-mTOR antibody	Cell Signaling	#2972, RRID: AB_330978
anti-Cpt1b antibody	Santa Cruz	sc-20522, RRID: AB_2084698
anti-Cpt1b antibody	Proteintech	22170-1-AP, RRID: AB_2713959
anti-Ucp1 antibody	Abcam	ab10983, RRID: AB_2241462
anti-Cre antibody	Cell Signaling	#12830, RRID: AB_2631055
anti-F4/80 antibody	Abcam	ab111101, RRID: AB_10859466
wheat germ agglutinin-conjugated Alexa Fluor 488	Thermo Fisher Scientific	W11261
Hoechst	Life Technologies	33258
horseradish peroxidase-conjugated anti-rabbit immunoglobulin G	Jackson Immunoresearch	#111-035-003, RRID: AB_2313567
donkey anti-goat IgG H&L (Cy5)	Abcam	ab6566, RRID: AB_955056
goat anti-rabbit IgG H&L (DyLight488)	Abcam	ab96899, RRID: AB_10679361
goat anti-rabbit IgG H&L (Cy5)		ab97077, RRID: AB_10679461
Biotin-SP (long spacer) AffiniPure Donkey Anti-Rabbit IgG (H + L)	Jackson Immunoresearch	711-065-152, RRID: AB_2340593
BB515 Streptavidin	BD	564453, RRID: AB_2869580
Cy5-streptavidin conjugate	Invitrogen	43-4316

**Bacterial and virus strains**		

AAV-DJ Helper Free shRNA Expression System	Cell Biolabs Inc	VPK-413-DJ
AAV-DJ Helper Free Expression System	Cell Biolabs Inc	VPK-410-DJ

**Chemicals, peptides, and recombinant proteins**		

L-methionine sulfone	WAKO	502-76641
MES	Dojindo	349-01623
CSA	WAKO	037-01032
NADH	Sigma-Aldrich	N7410-15VL
ADP	Sigma-Aldrich	A4386-1G
LDH	Sigma-Aldrich	L1254-1KU
PK	Sigma-Aldrich	P7768-1KU
Enolase	Sigma-Aldrich	E6126-500UN
2,3-DPG	Santa Cruz	SC-213964
3-PGA	Santa Cruz	sc-214793
Rapamycin	WAKO	550-83271
Leucine	Sigma-Aldrich	L8000
Isoleucine	Sigma-Aldrich	I2752
Valine	Sigma-Aldrich	V0500
MHY1485	Sigma-Aldrich	SML0810

**Critical commercial assays**		

Norepinephrine ELISA Kit	Abnova	KA1891
*In Situ* Cell Death Detection Kit, Fluorescein	Roche	1684795
Ultra Sensitive Mouse Insulin ELISA Kit	Morinaga	M1104
Branched Chain Amino Acid Kit	Sigma-Aldrich	MAK003
Agilent SurePrint G3 Mouse GE microarray (G4852A)	Agilent	G4852A

**Deposited data**		

DNA microarray	This paper	GSE186030
Single cell RNA-sequence	This paper	GSE200374

**Experimental models: Cell lines**		

NIH-3T3L1 cells	ATCC	CL-173

**Experimental models: Organisms/strains**		

Mouse: Adipoq-Cre^+^: *Pgam1*^flox/flox^	Adipoq-Cre; Eguchi J et al., 2011. floxed Pgam1; Generated in our lab.	N/A
Mouse: Ucp1-Cre^+^: *Pgam1*^flox/flox^	UCP1-Cre; Kong X et al., 2014. floxed Pgam1; Generated in our lab.	N/A

**Oligonucleotides**		

Forward primer for mouse *Actb*; 5′-CTAAGGCCAACCGTGAAAAG-3′	This paper	N/A
Reverse primer for mouse *Actb*; 5′-ACCAGAGGCATACAGGGACA-3′	This paper	N/A
Forward primer for mouse *Rplp0*; 5′-GATGCCCAGGGAAGACAG-3′	This paper	N/A
Reverse primer for mouse *Rplp0*; 5′-ACAATGAAGCATTTTGGATAA-3′	This paper	N/A
Forward primer for mouse *Pgam1*; 5′-GCCTGATCACCCCTTCTACA-3′	This paper	N/A
Reverse primer for mouse *Pgam1*; 5′-CTCTTTGATCTGGGGGACAA-3′	This paper	N/A
Forward primer for mouse *Pgam2*; 5′-GGCATTGTGAAACATCTGGAA-3′	This paper	N/A
Reverse primer for mouse *Pgam2*; 5′-AACCTCATGGGCTTGGTG-3′	This paper	N/A
Forward primer for mouse *Cpt1b*; 5′-GAGTGACTGGTGGGAAGAATATG-3′	This paper	N/A
Reverse primer for mouse *Cpt1b*; 5′-GCTGCTTGCACATTTGTGTT-3′	This paper	N/A
Forward primer for mouse *Ucp1*; 5′-GGCCTCTACGACTCAGTCCA-3′	This paper	N/A
Reverse primer for mouse *Ucp1*; 5′-TAAGCCGGCTGAGATCTTGT-3′	This paper	N/A
Forward primer for mouse *Tnfrsf9*; 5′-CGTCTGTCGACCCTGGAC-3′	This paper	N/A
Reverse primer for mouse *Tnfrsf9*; 5′-CACGTCCTTCTCCGTGGT-3′	This paper	N/A
Forward primer for mouse *Cidea* 5′-TTCAAGGCCGTGTTAAGGA-3′	This paper	N/A
Reverse primer for mouse *Cidea* 5′-CCTTTGGTGCTAGGCTTGG-3′	This paper	N/A
Forward primer for mouse *Egln3*; 5′-TGTCTGGTACTTCGATGCTGA-3′	This paper	N/A
Reverse primer for mouse *Egln3*; 5′-AGCAAGAGCAGATTCAGTTTTTC-3′	This paper	N/A
Forward primer for mouse *Kcnk3*; 5′-CCTTCTACTTCGCCATCACC-3′	This paper	N/A
Reverse primer for mouse *Kcnk3*; 5′-GCGTAGAACATGCAGAACACC-3′	This paper	N/A
Forward primer for mouse *Mtus1*; 5′-AAACACTGTCATTTTCCACACAG-3′	This paper	N/A
Reverse primer for mouse *Mtus1*; 5′-CTGGGTCTGGATGCATAGG-3′	This paper	N/A
Forward primer for mouse *Ppargc1a*; 5′-TGAAAGGGCCAAACAGAGAG-3′	This paper	N/A
Reverse primer for mouse *Ppargc1a*; 5′-GTAAATCACACGGCGCTCTT-3′	This paper	N/A
Forward primer for mouse *Prdm16*; 5′-ACAGGCAGGCTAAGAACCAG-3′	This paper	N/A
Reverse primer for mouse *Prdm16*; 5′-CGTGGAGAGGAGTGTCTTCAG-3′	This paper	N/A
Forward primer for mouse *Tbx1*; 5′-TTTGTGCCCGTAGATGACAA-3′	This paper	N/A
Reverse primer for mouse *Tbx1*; 5′-CTCGGCCAGGTGTAGCAG-3′	This paper	N/A
Forward primer for mouse *Tmem26*; 5′-CTGCTCAACCTCTTGCTGGT-3′	This paper	N/A
Reverse primer for mouse *Tmem26*; 5′-AAGATGGCCGGAGAAAGC-3′	This paper	N/A

**Recombinant DNA**		

Forward sequence for sh-mouse Pgam1; 5′-GATCCGGGCATCCCTATCGTCTATGATTCAAGAG ATCATAGACGATAGGGATGCCCTTTTTTACGCGTG-3′	This paper	N/A
Reverse sequence for sh-mouse Pgam1; 5′-AATTCACGCGTAAAAAAGGGCATCCCTATCGTCT ATGATCTCTTGAATCATAGACGATAGGGATGCCCG-3′	This paper	N/A

**Software and algorithms**		

SPSS version 24 software	IBM	N/A
KEGG identifiers	KEGG (Kyoto Encyclopedia of Genes and Genomes)	N/A

### Resource availability

#### Lead contact

Further information and requests for resources and reagents should be directed to and will be fulfilled by the Lead Contact, Tohru Minamino at t.minamino@juntendo.ac.jp.

#### Materials availability

This study did not generate new unique reagents.

### Experimental model and subject details

#### Animal models

All of the animal experiments were conducted in compliance with the protocol reviewed by the Institutional Animal Care and Use Committee of Niigata University and approved by the President of Niigata University. Adipoq-Cre mice (Eguchi et al., 2011) were purchased from the Jackson Laboratory (ME, U.S.A.). Adipoq-Cre or Ucp1-Cre (C57BL/6J background)(Kong et al., 2014) were crossed with mice carrying floxed *Pgam1* alleles with a C57BL/6 background to generate mice with adipose tissue- (Adipo-Pgam1 KO) or brown adipose tissue (BAT)- (Ucp1-Pgam1 KO) specific knockout of Pgam1. The genotypes of the littermate controls were Adipoq-Cre^–^: *Pgam1* ^flox/flox^ or Ucp1-Cre^–^: *Pgam1* ^flox/flox^. CAG-LSL-EGFP mice were provided by RIKEN (RBRC09806)(Kawamoto et al., 2000). All experiments were performed using male mice.

### Method details

#### Cell culture

The mouse pre-adipocyte cell line, NIH-3T3L1 cells were cultured in high glucose DMEM (Gibco, 12430) with 10% fetal bovine serum (FBS) and 100U/mL penicillin/streptomycin solution (P/S), and differentiation was induced by adding 0.5 mM 3-isobutyl-1-methylxanthine, 1 mM dexamethasone, and 5 mg/mL insulin (Sigma Aldrich) for 48 h. Fully differentiated adipocytes were used for further analysis after 7 days of culture. In some experiments, short hairpin RNA (shRNA) targeting mouse Pgam1 (shPgam1), negative control (shCont) or mouse Bcat1 cDNA were introduced to NIH-3T3L1 cells by using adenovirus-assocoated vector (AAV) as described below. In some experiments, fully differentiated adipocytes were treated with rapamycin (WAKO, 550-83,271), leucine (Sigma-Aldrich, L8000), isoleucine (Sigma-Aldrich, I2752), valine (Sigma-Aldrich, V0500) or MHY1485 (Sigma-Aldrich, SML0810). In some experiments, adipocytes were cultured in DMEM without branched chain amino acids (BCAA; valine, leucine and isoleucine) (Gmep inc., Kurume, Japan).

#### Adeno associated virus (AAV)

pAAV-sh-*Pgam1* and pAAV-sh-Negative control vectors were constructed using standard subcloning techniques according to the manufacturer’s instructions (AAV-DJ Helper Free shRNA Expression System, Cell Biolabs Inc, VPK-413-DJ). Annealed complementary nucleotides for shRNA target site against mouse *Pgam1* or negative control were subcloned into *Bam*HI/*Eco*RI sites of pAAV-U6-GFP expression vector. We co-transfected HEK293 cells with pAAV expression vector, pAAV-DJ and pHelper using transfection reagent (X-tremeGENE9 DNA Transfection Reagent, Roche, 06365809001). AAV was harvested from these cells by freeze and thaw cycles, and purified with a ViraBind AAV Purification Kit (Cell Biolabs Inc, VPK-140). Titer of purified AAV was quantified with QuickTiter AAV Quantitation Kit (Cell Biolabs Inc, VPK-145). We added AAV into NIH-3T3L1 cells with a titer of 1 × 10ˆ9 GC/well. Sequence of shRNA targeting mouse *Pgam1* were as follows.

sh-Pgam1

5′-GATCCGGGCATCCCTATCGTCTATGATTCAAGAGATCATAGACGATAGGGATGCCCTTTTTTACGCGTG-3′

5′-AATTCACGCGTAAAAAAGGGCATCCCTATCGTCTATGATCTCTTGAATCATAGACGATAGGGATGCCCG-3′

#### Physiological analyses

Mice were housed individually for monitoring of their body weight and food intake. Adiposity was examined by CT scanning (LaTheta, Aloca) according to the manufacturer’s protocol. CT scans were obtained at 2-mm intervals from the diaphragm to the base of the abdominal cavity. Oxygen consumption was measured in 20-week-old mice by using an O2/CO2 metabolic measurement system (Columbus Instruments) according to the manufacturer’s instruction.

#### Laboratory tests

Experiments were done in all animal models at 12 weeks of age unless otherwise mentioned. Mice were housed individually for one week prior to the assay. On the day of the glucose tolerance test (GTT), the mice were fasted for 6 h and then glucose was injected intraperitoneally at a dose of 2 g/kg in the early afternoon. Blood glucose levels were measured with a glucose analyzer (SANWA KAGAKU KENKYUSHO) at 15, 30, 60, and 120 min after glucose injection. For the insulin tolerance test (ITT), mice were given human insulin intraperitoneally (1 U/kg body weight) at 1:00 p.m. without starvation. Tail vein blood was collected at 0, 15, 30, 60, and 120 min after administration, and blood glucose levels were measured with a glucose analyzer. We measured the levels of insulin by using ELISA-based immunoassay kits (Morinaga Institute of Biological Science Inc.) according to the manufacturer’s instruction. Norepinephrine levels in plasma and gWAT were measured by enzyme-linked immunosorbent assay (ELISA) (Abnova, Walnut CA, USA, KA1891) according to the manufacturer’s instructions.

#### Acute cold exposure

Body temperature was assessed by subcutaneous implantation of biocompatible and sterile microchip transponders (IPTT-300 Extended Accuracy Calibration; Bio Medic Data Systems) in the scapular region according to the manufacturer’s instructions. Animals were subjected to the cold tolerance test (CTT) at 4°C and body temperature was measured at hourly intervals for 6–8 h.

#### Metabolomic analyses

Metabolomic analyses were done by Soga et al. at Keio University using capillary electrophoresis time-of-flight/mass spectrometry (CE-TOF/MS), as described previously (Hirayama et al., 2009). Interscapular BAT, gonadal WAT (gWAT) and inguinal WAT (iWAT) samples from Adipo-Pgam1 KO mice were corrected from the mice and immediately frozen in liquid nitrogen. To extract metabolites, 40–50mg snap-frozen samples were completely homogenized by Shake Master *NEO* (Bio Medical Science) in 500uL of methanol containing L-methionine sulfone (Wako 502-76641), methionine sulfone (MES, Dojindo 349-01623), and D-camphor-10-sulfonic acid sodium salt (CSA, Wako 037-01032) (all at 20 μM). After adding CHCl3 (500 μL) and distilled water (200 μL) with thorough mixing, centrifugation was performed at 4,600g for 15 min at 4°C. Then the aqueous layer (300 μL) was collected and transferred to a 5-kDa cutoff filter (UltrafreeMC-PLHCC; Human Metabolome Technologies, UFC3LCCNB-HMT). Centrifugation was performed at 9,100g for 3 h at 20°C, after which the filtrate was centrifugally concentrated and dissolved in 50μL distilled water containing reference compounds (200 μM each of 3-aminopyrrolidine and trimesate). Samples were analyzed by capillary electrophoresis time-of-flight/mass spectrometry (CE-TOF/MS). All CE-TOF/MS experiments were performed using an Agilent CE Capillary Electrophoresis System equipped with an Agilent TOFMS, an Agilent 1100 isocratic HPLC pump, an Agilent G1603A CE-MS adapter kit, and an Agilent G1607A CE–electrospray ionization (ESI)–MS sprayer kit (Agilent Technologies). For system control and data acquisition, we used Agilent G2201AA ChemStation software for CE and Agilent TOF (Analyst QS) for TOFMS. Anionic metabolites were separated in a cationic-polymer–coated COSMO(+) capillary (50 μm i.d. 100 cm total length) (Nacalai Tesque) filled with 50 mmol/L ammonium acetate solution (pH 8.5) as the running electrolyte. Sample was injected with a pressure injection of 50 mbar for 30 s (approximately 30 nL) and a negative voltage of 30kV was applied. Ammonium acetate (5 mmol/L) in 50% methanol/water (50% v/v) containing 0.01 μM Hexakis (2,2-difluoroethoxy) phosphazene was delivered as the sheath liquid at 10 μL/min. ESI-TOFMS was conducted in the negative ionization. The capillary voltage was set at 3500V. For TOFMS, the fragmenter voltage, skimmer voltage, and OCT RFV were set at 100, 50, and 500 V, respectively. A flow rate of drying nitrogen gas (heater temper-ature, 300°C) was maintained at 7 L/min. Automatic recalibration of each acquired spectrum was performed using reference masses of reference standards ([13C isotopic ion of deprotonated acetic acid dimer (2CH3COOH - H)]-, *m*/*z* 120.03841), and ([Hexakis + deprotonated acetic acid (CH3COOH - H)]-, *m*/*z* 680.03554). Exact mass data were acquired at a rate of 1.5 spectra/s over a 50-1000 *m*/*z* range. Cationic metabolites were separated in a fused-silica capillary (50 μm i.d. 100 cm total length) filled with 1 M formic acid as the runnning electrolyte. Sample was injected with a pressure injection of 50 mbar for 5 s (approximately 5 nL), and positive voltage of 30 kV was applied. Sheath liquid that comprised methanol/water (50% v/v) containing 0.01 μM Hexakis (2,2-difluoroethoxy) phosphazene was delivered at 10 μL/min. ESI-TOFMS was operated in the positive ionization. The capillary voltage was set at 4000V. For TOFMS, the fragmenter voltage, skimmer voltage, and OCT RFV were set at 75, 50, and 500 V, respectively. Automatic recalibration of each acquired spectrum was performed using reference masses of reference standards ([2MeOH13Cisotope]-, *m*/*z* 66.06306), and ([Hexakis(2,2-difluoroethoxy)phosphazene]-, *m*/*z* 622.028963). Exact mass data were acquired at the rate of 1.5 cycles/s over a 50 to 1,000 *m*/*z* range. Raw CE-TOFMS data were processed using Master Hands ver. 2.17.0.8 software for the quantification of metabolites. Similar results were obtained when metabolite levels were adjusted by total protein or tissue weight.

#### Microarray analyses

Total RNA was isolated from BAT, gWAT and iWAT of Adipo-Pgam1 KO with RNeasy Lipid Tissue Kit (QIAGEN). Cyanine-3 (Cy3) labeled cRNA was prepared from 600 ng RNA using the One-Color Microarray-Based Gene Expression Analysis (Low Input Quick Amp Labeling) (Agilent, CA, USA) according to the manufacturer’s instructions, and the resulting probes were hybridized to Agilent SurePrint G3 Mouse GE Microarray kit (8 × 60K) (G4852B). The scanned images were normalized by Agilent GeneSpring GX 12.0 software and differentially expressed genes were identified via the fold-change (FC) and p values of the *t*-test. Gene expression data are available through the Gene Expression Omnibus database (GSE186030).

#### scRNA-seq analysis

scRNA-seq analysis was done at University of Tokyo using Illumina HiSeq3000 sequencer. For isolation of the stromal vascular cell fraction (SVF) from adipose tissue, gWAT was excised, minced, and placed into 10 mL of PBS(−) containing 2 mg/mL of type 2 collagenase (Worthington, Lakewood, NJ), 1.2 U/mL of Dispase (Gibco, Carlsbad, CA), and 10 mM CaCl2 (Roche). Incubation was done for 20 min at 37°C with gentle agitation. After adding 10 mL of DMEM with 10% FBS, the lysate was centrifuged at 400×g for 5 min and the pellet was resuspended in 5 mL of erythrocyte lysis buffer and incubated for 5 min at room temperature. Then the lysate was filtered through a cell strainer (70 μm) into a 50 mL tube. Next, the lysate was centrifuged at 400×g for 5 min and the pellet was resuspended in 5 mL of PBS and filtered through a cell strainer (40 μm) into a 50 mL tube. The SVF cells were centrifuged at 400×g and resuspended in 500 μL of PBS for further analysis. The scRNA-seq libraries was prepared using Chromium Single Cell 3ʹ Reagent Kits v3 and Chromium controller (10x Genomics). 28/96 bp pair end sequencing of the libraries was performed by Illumina HiSeq3000. Using CellRanger v3.0.1, the sequencing data was processed, and the count matrices were generated. Data analysis of count matrices was performed by Seurat (version 3.1.5) on R (version 4.0.1) and R studio (version 1.1.463). Gene expression data are available through the Gene Expression Omnibus database (GSE200374).

#### Histological analyses and TEM study

Interscapular BAT, gWAT and iWAT samples were harvested from mice, fixed overnight in 10% formalin, embedded in paraffin, and sectioned for immunofluorescence or hematoxylin-eosin (HE) staining. The following antibodies were used: anti-Cpt1b antibody (Santa Cruz, sc-20522), anti-Cpt1b antibody (Proteintech, 22170-1-AP), anti-Ucp1 antibody (Abcam, ab10989), anti-Cre antibody (Cell Signaling, #12830), anti-F4/80 antibody (Abcam, ab111101), wheat germ agglutinin-conjugated Alexa Fluor 488 for staining cell membranes (Thermo Fisher Scientific, W11261), and Hoechst (Life Technologies, 33258). The secondary antibody for anti-Cpt1b antibody (Santa Cruz, sc-20522) was donkey anti-goat IgG H&L (Cy5) (Abcam, ab6566), while that for Ucp1 antibody was goat anti-rabbit IgG H&L (DyLight488) (Abcam, ab96899). The secondary antibody for anti-Cpt1b antibody (Proteintech, 22170-1-AP) and anti-F4/80 antibody was goat anti-rabbit IgG H&L (Cy5) (Abcam, ab97077). The secondary antibody for anti-Cre antibody was Biotin-SP (long spacer) AffiniPure Donkey Anti-Rabbit IgG (H + L) (Jackson Immunoresearch, 711-065-152) followed by BB515 Streptavidin (BD, 564453). All primary and secondary antibodies were used at a dilution of 1:50, except for Hoechst (1:1000). Stained sections were photographed with a Biorevo (Keyence Co.) or a confocal microscopy (C2+, Nicon Co.). Immunostaining was quantified with ImageJ software, and the UCP1- or CPT1b-positive area was calculated as the ratio of the DyLight488-or Cy5-positive area to the whole area of each view. For electron microscopy, interscapular BAT and gWAT were fixed in 2.5% glutaraldehyde/2.0% paraformaldehyde in 0.1M cacodylate buffer. Then 50 mg of calcium chloride was added to 400 mL of fixative. Grids for electron microscopy were prepared by Masaaki Nameta at the electron microscope core facility of Niigata University, and electron microscopy was performed by using a JEM1400 TEM at Niigata University Medical Campus.

#### RNA analysis

Total RNA (1 μg) was isolated from tissue samples with RNA-Bee (TEL-TEST Inc.). Real-time PCR (qPCR) was performed by using a Light Cycler 480 (Roche) with the Universal Probe Library and the Light Cycler 480 Probes Master (Roche) or LightCycler 480 SYBR Green I Master (Roche) according to the manufacturer’s instructions. The primers and their sequences were as follows. *Actb* or *Rplp0* was used as the internal control.

*Actb*; 5′-CTAAGGCCAACCGTGAAAAG-3′, 5′-ACCAGAGGCATACAGGGACA-3′

*Rplp0* 5′-GATGCCCAGGGAAGACAG-3′, 5′-ACAATGAAGCATTTTGGATAA-3′

*Pgam1*; 5′-GCCTGATCACCCCTTCTACA-3′, 5′-CTCTTTGATCTGGGGGACAA-3′

*Pgam2*; 5′-GGCATTGTGAAACATCTGGAA-3′, 5′-AACCTCATGGGCTTGGTG-3′

*Cpt1b*; 5′-GAGTGACTGGTGGGAAGAATATG-3′, 5′-GCTGCTTGCACATTTGTGTT-3′

*Ucp1*; 5′-GGCCTCTACGACTCAGTCCA-3′, 5′-TAAGCCGGCTGAGATCTTGT-3′

*Tnfrsf9*; 5′-CGTCTGTCGACCCTGGAC-3′, 5′-CACGTCCTTCTCCGTGGT-3′

*Cidea*; 5′-TTCAAGGCCGTGTTAAGGA-3′, 5′-CCTTTGGTGCTAGGCTTGG-3′

*Egln3*; 5′-TGTCTGGTACTTCGATGCTGA-3′, 5′-AGCAAGAGCAGATTCAGTTTTTC-3′

*Kcnk3*; 5′-CCTTCTACTTCGCCATCACC-3′, 5′-GCGTAGAACATGCAGAACACC-3′

*Mtus1*; 5′-AAACACTGTCATTTTCCACACAG-3′, 5′-CTGGGTCTGGATGCATAGG-3′

*Ppargc1a*; 5′-TGAAAGGGCCAAACAGAGAG-3′, 5′-GTAAATCACACGGCGCTCTT-3′

*Prdm16*; 5′-ACAGGCAGGCTAAGAACCAG-3′, 5′-CGTGGAGAGGAGTGTCTTCAG-3′

*Tbx1*; 5′-TTTGTGCCCGTAGATGACAA-3′, 5′-CTCGGCCAGGTGTAGCAG-3′

*Tmem26*; 5′-CTGCTCAACCTCTTGCTGGT-3′, 5′-AAGATGGCCGGAGAAAGC-3′

#### Western blot analysis

Whole-cell lysates were prepared in lysis buffer (10 mM Tris-HCl, pH 8, 140 mM NaCl, 5 mM EDTA, 0.025% NaN3, 1% Triton X-100, 1% deoxycholate, 0.1% SDS, 1 mM PMSF, 5 μg ml–1 leupeptin, 2 μg ml–1 aprotinin, 50 mM NaF, and 1 mM Na_2_VO_3_). Then the lysates (40–50 μg) were resolved by SDS-PAGE. Proteins were transferred to a PVDF membrane (Millipore) that was incubated with the primary antibody, followed by incubation with horseradish peroxidase-conjugated anti-rabbit immunoglobulin G (Jackson Immunoresearch). Proteins were detected by enhanced chemiluminescence (GE). The primary antibodies for western blotting were anti-Pgam1 antibody (Abcam, ab2220), anti-actin antibody (Cell Signaling, #4970), anti-GAPDH (Cell Signaling, #2118), anti-Tubulin (Cell Signaling, #2125), anti-p62 (Abcam, ab219581), and anti-phospho mTOR (Cell Signaling, #2971), and anti-mTOR (Cell Signaling, #2972). The primary antibodies were used at a dilution of 1:5000 for anti-actin and anti-GAPDH antibody, 1:1000 for anti-Pgam1, anti-Tubulin, anti-p62, anti-phospho mTOR, and anti-mTOR antibody.

#### PGAM enzymatic activity assay

PGAM enzymatic activity was measured as previously described (Hallows et al., 2012) with as slight modification. Briefly, adipose tissue lysates were prepared with lysis buffer (10 mM Tris-HCl (pH 7.8), 1% NP-40, 150 mM NaCl and 1mM EDTA). The reaction mixture contained 100 mM Tris-HCl, pH 8.0, 0.5 mM EDTA, 2 mM MgCl2, 100 mM KCl, 0.2 mM NADH, 3 mM ADP, 10 μM 2,3-bisphosphoglycerate, lactate dehydrogenase (LDH) (600 munit/mL), pyruvate kinase (500 munit/mL), enolase (100 munit/mL), and 1 mM 3-phosphoglycerate. Assays were performed at 37°C and measured at 340 nm using the FilterMax 5 microplate reader (Molecular Devices).

#### BCAA intercellular concentration assay

This assay using Branched Chain Amino Acid Kit (Sigma-Aldrich, #MAK003), and all processes were constructed according to the manufacturer’s instructions. After differentiated NIH3T3L1 cells infected AAV shPgam1, and rapidly homogenized in cold BCAA Assay Buffer. Collect the supernatant and adjust to the same volume by BCAA Assay Buffer after centrifuge. The supernatant was added to mixed appropriate Reaction Mix included BCAA Enzyme Mix and WST Substrate Mix into 96 well plates and incubated the reaction for 30 min at room temperature (blank mixed Reaction Mix without BCAA Enzyme Mix), and measured the absorbance at 450 nm (A_450_). Intercellular BCAA concentration was normalized with BCAA standard, which is provided from kit, and determined the amount of BCAA presented in the sample from the standard curve.

### Quantification and statistical analysis

#### Enrichment analysis

Genes in the microarray analysis were mapped to KEGG identifiers defined in the KEGG (Kyoto Encyclopedia of Genes and Genomes) database (downloaded on May 21, 2014) (Kanehisa et al., 2021). The number of genes with a 1.2-fold or greater change in each KEGG PATHWAY category was counted. All the genes in the mouse genome were used as the reference set for the enrichment analysis. The KEGG PATHWAY categories that were significantly enriched were extracted based on q-values (Storey and Tibshirani, 2003) from Fisher’s exact test performed using R (http://www.r-project.org/).

#### Statistical analysis

Statistical analyses were done with SPSS version 24 software. Data are shown as the mean ± SEM Differences between groups were examined by the two-tailed Student’s *t* test or two-way ANOVA, followed by Tukey’s multiple comparison test, the non-parametric Kruskal Wallis test, or Dunnett’s test for comparisons among more than two groups. In ITT, GTT, and CTT, data was analyzed with repeated measures. In all analyses, p < 0.05 was considered statistically significant. Asterisks ∗ and ∗∗ were defined as p < 0.05 and p < 0.01, respectively.

## Data Availability

All data reported in this paper will be shared by the Lead contact upon request. Gene expression data are available through the Gene Expression Omnibus database (GSE186030, GSE200374).

## References

[bib1] Altshuler-Keylin S., Shinoda K., Hasegawa Y., Ikeda K., Hong H., Kang Q., Yang Y., Perera R.M., Debnath J., Kajimura S. (2016). Beige adipocyte maintenance is regulated by autophagy-induced mitochondrial clearance. Cell Metab..

[bib2] Bartelt A., Bruns O.T., Reimer R., Hohenberg H., Ittrich H., Peldschus K., Kaul M.G., Tromsdorf U.I., Weller H., Waurisch C. (2011). Brown adipose tissue activity controls triglyceride clearance. Nat. Med..

[bib3] Bi P., Shan T., Liu W., Yue F., Yang X., Liang X.R., Wang J., Li J., Carlesso N., Liu X., Kuang S. (2014). Inhibition of Notch signaling promotes browning of white adipose tissue and ameliorates obesity. Nat. Med..

[bib4] Bordicchia M., Liu D., Amri E.Z., Ailhaud G., Dessi-Fulgheri P., Zhang C., Takahashi N., Sarzani R., Collins S. (2012). Cardiac natriuretic peptides act via p38 MAPK to induce the brown fat thermogenic program in mouse and human adipocytes. J. Clin. Invest..

[bib5] Bostrom P., Wu J., Jedrychowski M.P., Korde A., Ye L., Lo J.C., Rasbach K.A., Bostrom E.A., Choi J.H., Long J.Z. (2012). A PGC1-alpha-dependent myokine that drives brown-fat-like development of white fat and thermogenesis. Nature.

[bib6] Crane J.D., Palanivel R., Mottillo E.P., Bujak A.L., Wang H., Ford R.J., Collins A., Blumer R.M., Fullerton M.D., Yabut J.M. (2015). Inhibiting peripheral serotonin synthesis reduces obesity and metabolic dysfunction by promoting brown adipose tissue thermogenesis. Nat. Med..

[bib7] Cypess A.M., Lehman S., Williams G., Tal I., Rodman D., Goldfine A.B., Kuo F.C., Palmer E.L., Tseng Y.H., Doria A. (2009). Identification and importance of brown adipose tissue in adult humans. N. Engl. J. Med..

[bib8] Cypess A.M., White A.P., Vernochet C., Schulz T.J., Xue R., Sass C.A., Huang T.L., Roberts-Toler C., Weiner L.S., Sze C. (2013). Anatomical localization, gene expression profiling and functional characterization of adult human neck brown fat. Nat. Med..

[bib9] Eguchi J., Wang X., Yu S., Kershaw E.E., Chiu P.C., Dushay J., Estall J.L., Klein U., Maratos-Flier E., Rosen E.D. (2011). Transcriptional control of adipose lipid handling by IRF4. Cell Metab..

[bib10] Fisher F.M., Kleiner S., Douris N., Fox E.C., Mepani R.J., Verdeguer F., Wu J., Kharitonenkov A., Flier J.S., Maratos-Flier E., Spiegelman B.M. (2012). FGF21 regulates PGC-1α and browning of white adipose tissues in adaptive thermogenesis. Genes Dev..

[bib11] Gnad T., Scheibler S., von Kugelgen I., Scheele C., Kilic A., Glode A., Hoffmann L.S., Reverte-Salisa L., Horn P., Mutlu S. (2014). Adenosine activates brown adipose tissue and recruits beige adipocytes via A2A receptors. Nature.

[bib12] Halama A., Horsch M., Kastenmuller G., Moller G., Kumar P., Prehn C., Laumen H., Hauner H., Hrabe de Angelis M., Beckers J. (2016). Metabolic switch during adipogenesis: from branched chain amino acid catabolism to lipid synthesis. Arch. Biochem. Biophys..

[bib13] Hallows W.C., Yu W., Denu J.M. (2012). Regulation of glycolytic enzyme phosphoglycerate mutase-1 by Sirt1 protein-mediated deacetylation. J. Biol. Chem..

[bib14] Hankir M.K., Cowley M.A., Fenske W.K. (2016). A BAT-centric approach to the treatment of diabetes: turn on the brain. Cell Metab..

[bib15] Hankir M.K., Klingenspor M. (2018). Brown adipocyte glucose metabolism: a heated subject. EMBO Rep..

[bib16] Harms M., Seale P. (2013). Brown and beige fat: development, function and therapeutic potential. Nat. Med..

[bib17] Hirayama A., Kami K., Sugimoto M., Sugawara M., Toki N., Onozuka H., Kinoshita T., Saito N., Ochiai A., Tomita M. (2009). Quantitative metabolome profiling of colon and stomach cancer microenvironment by capillary electrophoresis time-of-flight mass spectrometry. Cancer Res..

[bib18] Hitosugi T., Zhou L., Elf S., Fan J., Kang H.B., Seo J.H., Shan C., Dai Q., Zhang L., Xie J. (2012). Phosphoglycerate mutase 1 coordinates glycolysis and biosynthesis to promote tumor growth. Cancer Cell.

[bib19] Hui X., Gu P., Zhang J., Nie T., Pan Y., Wu D., Feng T., Zhong C., Wang Y., Lam K.S., Xu A. (2015). Adiponectin enhances cold-induced browning of subcutaneous adipose tissue via promoting M2 macrophage proliferation. Cell Metab..

[bib20] Ikegami R., Shimizu I., Sato T., Yoshida Y., Hayashi Y., Suda M., Katsuumi G., Li J., Wakasugi T., Minokoshi Y. (2018). Gamma-aminobutyric acid signaling in Brown adipose tissue promotes systemic metabolic derangement in obesity. Cell Rep..

[bib21] Isidor M.S., Winther S., Markussen L.K., Basse A.L., Quistorff B., Nedergaard J., Emanuelli B., Hansen J.B. (2020). Pyruvate kinase M2 represses thermogenic gene expression in brown adipocytes. FEBS Lett..

[bib22] Ji S., You Y., Kerner J., Hoppel C.L., Schoeb T.R., Chick W.S., Hamm D.A., Daniel Sharer J., Wood P.A. (2008). Homozygous carnitine palmitoyltransferase 1b (muscle isoform) deficiency is lethal in the mouse. Mol. Genet. Metab..

[bib23] Jiang X., Sun Q., Li H., Li K., Ren X. (2014). The role of phosphoglycerate mutase 1 in tumor aerobic glycolysis and its potential therapeutic implications. Int. J. Cancer.

[bib24] Jung S.M., Doxsey W.G., Le J., Haley J.A., Mazuecos L., Luciano A.K., Li H., Jang C., Guertin D.A. (2021). In vivo isotope tracing reveals the versatility of glucose as a brown adipose tissue substrate. Cell Rep..

[bib25] Kajimura S., Spiegelman B.M., Seale P. (2015). Brown and beige fat: physiological roles beyond heat generation. Cell Metab..

[bib26] Kanehisa M., Furumichi M., Sato Y., Ishiguro-Watanabe M., Tanabe M. (2021). KEGG: integrating viruses and cellular organisms. Nucleic Acids Res..

[bib27] Kawamoto S., Niwa H., Tashiro F., Sano S., Kondoh G., Takeda J., Tabayashi K., Miyazaki J.I. (2000). A novel reporter mouse strain that expresses enhanced green fluorescent protein upon Cre-mediated recombination. FEBS Lett..

[bib28] Kir S., Komaba H., Garcia A.P., Economopoulos K.P., Liu W., Lanske B., Hodin R.A., Spiegelman B.M. (2016). PTH/PTHrP receptor mediates cachexia in models of kidney failure and cancer. Cell Metab..

[bib29] Kondoh H., Lleonart M.E., Gil J., Wang J., Degan P., Peters G., Martinez D., Carnero A., Beach D. (2005). Glycolytic enzymes can modulate cellular life span. Cancer Res..

[bib30] Kong X., Banks A., Liu T., Kazak L., Rao R.R., Cohen P., Wang X., Yu S., Lo J.C., Tseng Y.H. (2014). IRF4 is a key thermogenic transcriptional partner of PGC-1α. Cell.

[bib31] Lee P., Smith S., Linderman J., Courville A.B., Brychta R.J., Dieckmann W., Werner C.D., Chen K.Y., Celi F.S. (2014). Temperature-acclimated brown adipose tissue modulates insulin sensitivity in humans. Diabetes.

[bib32] Lee J., Ellis J.M., Wolfgang M.J. (2015). Adipose fatty acid oxidation is required for thermogenesis and potentiates oxidative stress-induced inflammation. Cell Rep..

[bib33] Lin J.Z., Martagon A.J., Cimini S.L., Gonzalez D.D., Tinkey D.W., Biter A., Baxter J.D., Webb P., Gustafsson J.A., Hartig S.M., Phillips K. (2015). Pharmacological activation of Thyroid hormone receptors elicits a functional conversion of white to brown fat. Cell Rep..

[bib34] Ma X., Xu L., Alberobello A.T., Gavrilova O., Bagattin A., Skarulis M., Liu J., Finkel T., Mueller E. (2015). Celastrol protects against obesity and metabolic dysfunction through activation of a HSF1-PGC1α transcriptional axis. Cell Metab..

[bib35] Nagano G., Ohno H., Oki K., Kobuke K., Shiwa T., Yoneda M., Kohno N. (2015). Activation of classical brown adipocytes in the adult human perirenal depot is highly correlated with PRDM16-EHMT1 complex expression. PLoS One.

[bib36] Nguyen H.P., Yi D., Lin F., Viscarra J.A., Tabuchi C., Ngo K., Shin G., Lee A.Y.f., Wang Y., Sul H.S. (2020). Aifm2, a NADH oxidase, supports robust glycolysis and is required for cold- and diet-induced thermogenesis. Mol. Cell.

[bib37] Nie C., He T., Zhang W., Zhang G., Ma X. (2018). Branched chain amino acids: beyond nutrition metabolism. Int. J. Mol. Sci..

[bib38] Reitman M.L. (2017). How does fat transition from white to beige?. Cell Metab..

[bib39] Rogers N.H., Landa A., Park S., Smith R.G. (2012). Aging leads to a programmed loss of brown adipocytes in murine subcutaneous white adipose tissue. Aging Cell.

[bib40] Schreiber R., Diwoky C., Schoiswohl G., Feiler U., Wongsiriroj N., Abdellatif M., Kolb D., Hoeks J., Kershaw E.E., Sedej S. (2017). Cold-induced thermogenesis depends on ATGL-mediated lipolysis in cardiac muscle, but not brown adipose tissue. Cell Metab..

[bib41] Seale P., Conroe H.M., Estall J., Kajimura S., Frontini A., Ishibashi J., Cohen P., Cinti S., Spiegelman B.M. (2011). Prdm16 determines the thermogenic program of subcutaneous white adipose tissue in mice. J. Clin. Invest..

[bib58] Sharp L.Z., Shinoda K., Ohno H., Scheel D.W., Tomoda E., Ruiz L., Hu H., Wang L., Pavlova Z., Gilsanz V., Kajimura S. (2012). Human BAT possesses molecular signatures that resemble beige/brite cells. PLoS One.

[bib42] Shimizu I., Aprahamian T., Kikuchi R., Shimizu A., Papanicolaou K.N., MacLauchlan S., Maruyama S., Walsh K. (2014). Vascular rarefaction mediates whitening of brown fat in obesity. J. Clin. Invest..

[bib43] Shin H., Ma Y., Chanturiya T., Cao Q., Wang Y., Kadegowda A.K.G., Jackson R., Rumore D., Xue B., Shi H. (2017). Lipolysis in Brown adipocytes is not essential for cold-induced thermogenesis in mice. Cell Metab..

[bib59] Shinoda K., Luijten I.H., Hasegawa Y., Hong H., Sonne S.B., Kim M., Xue R., Chondronikola M., Cypess A.M., Tseng Y.H. (2015). Genetic and functional characterization of clonally derived adult human brown adipocytes. Nature medicine.

[bib44] Storey J.D., Tibshirani R. (2003). Statistical significance for genomewide studies. Proc. Natl. Acad. Sci. U S A.

[bib45] Tai T.A., Jennermann C., Brown K.K., Oliver B.B., MacGinnitie M.A., Wilkison W.O., Brown H.R., Lehmann J.M., Kliewer S.A., Morris D.C., Graves R.A. (1996). Activation of the nuclear receptor peroxisome proliferator-activated receptor gamma promotes brown adipocyte differentiation. J. Biol. Chem..

[bib46] Tan C.Y., Virtue S., Bidault G., Dale M., Hagen R., Griffin J.L., Vidal-Puig A. (2015). Brown adipose tissue thermogenic capacity is regulated by Elovl6. Cell Rep..

[bib47] Tolwani R.J., Hamm D.A., Tian L., Sharer J.D., Vockley J., Rinaldo P., Matern D., Schoeb T.R., Wood P.A. (2005). Medium-chain acyl-CoA dehydrogenase deficiency in gene-targeted mice. PLoS Genet..

[bib48] van der Lans A.A., Hoeks J., Brans B., Vijgen G.H., Visser M.G., Vosselman M.J., Hansen J., Jorgensen J.A., Wu J., Mottaghy F.M. (2013). Cold acclimation recruits human brown fat and increases nonshivering thermogenesis. J. Clin. Invest..

[bib49] Van Gaal L.F., Mertens I.L., De Block C.E. (2006). Mechanisms linking obesity with cardiovascular disease. Nature.

[bib50] Vegiopoulos A., Muller-Decker K., Strzoda D., Schmitt I., Chichelnitskiy E., Ostertag A., Berriel Diaz M., Rozman J., Hrabe de Angelis M., Nusing R.M. (2010). Cyclooxygenase-2 controls energy homeostasis in mice by de novo recruitment of brown adipocytes. Science.

[bib51] Villarroya F., Vidal-Puig A. (2013). Beyond the sympathetic tone: the new brown fat activators. Cell Metab..

[bib52] Whittle A.J., Carobbio S., Martins L., Slawik M., Hondares E., Vazquez M.J., Morgan D., Csikasz R.I., Gallego R., Rodriguez-Cuenca S. (2012). BMP8B increases brown adipose tissue thermogenesis through both central and peripheral actions. Cell.

[bib53] Wu J., Bostrom P., Sparks L.M., Ye L., Choi J.H., Giang A.H., Khandekar M., Virtanen K.A., Nuutila P., Schaart G. (2012). Beige adipocytes are a distinct type of thermogenic fat cell in mouse and human. Cell.

[bib54] Xue R., Lynes M.D., Dreyfuss J.M., Shamsi F., Schulz T.J., Zhang H., Huang T.L., Townsend K.L., Li Y., Takahashi H. (2015). Clonal analyses and gene profiling identify genetic biomarkers of the thermogenic potential of human brown and white preadipocytes. Nat. Med..

[bib55] Yanovski S.Z., Yanovski J.A. (2002). Obesity. N. Engl. J. Med..

[bib56] Yoneshiro T., Aita S., Matsushita M., Okamatsu-Ogura Y., Kameya T., Kawai Y., Miyagawa M., Tsujisaki M., Saito M. (2011). Age-related decrease in cold-activated brown adipose tissue and accumulation of body fat in healthy humans. Obesity (Silver Spring).

[bib57] Yoneshiro T., Aita S., Matsushita M., Kayahara T., Kameya T., Kawai Y., Iwanaga T., Saito M. (2013). Recruited brown adipose tissue as an antiobesity agent in humans. J. Clin. Invest..

