# Supplementary material for: Differing impact of phosphoglycerate mutase 1-deficiency on brown and white adipose tissue
Source: iScience. 2022 Apr 18;25(5):104268. doi: 10.1016/j.isci.2022.104268 (PMC9065309; doi:10.1016/j.isci.2022.104268)
Supplement: Document S1. Figures S1–S8 [file mmc1.pdf]

iScience, Volume *25*

Supplemental information

Differing impact of phosphoglycerate mutase 1-deficiency on brown and white adipose tissue

Yohko Yoshida, Ippei Shimizu, Yung-Ting Hsiao, Masayoshi Suda, Goro Katsuumi, Masahide Seki, Yutaka Suzuki, Shujiro Okuda, Tomoyoshi Soga, and Tohru Minamino

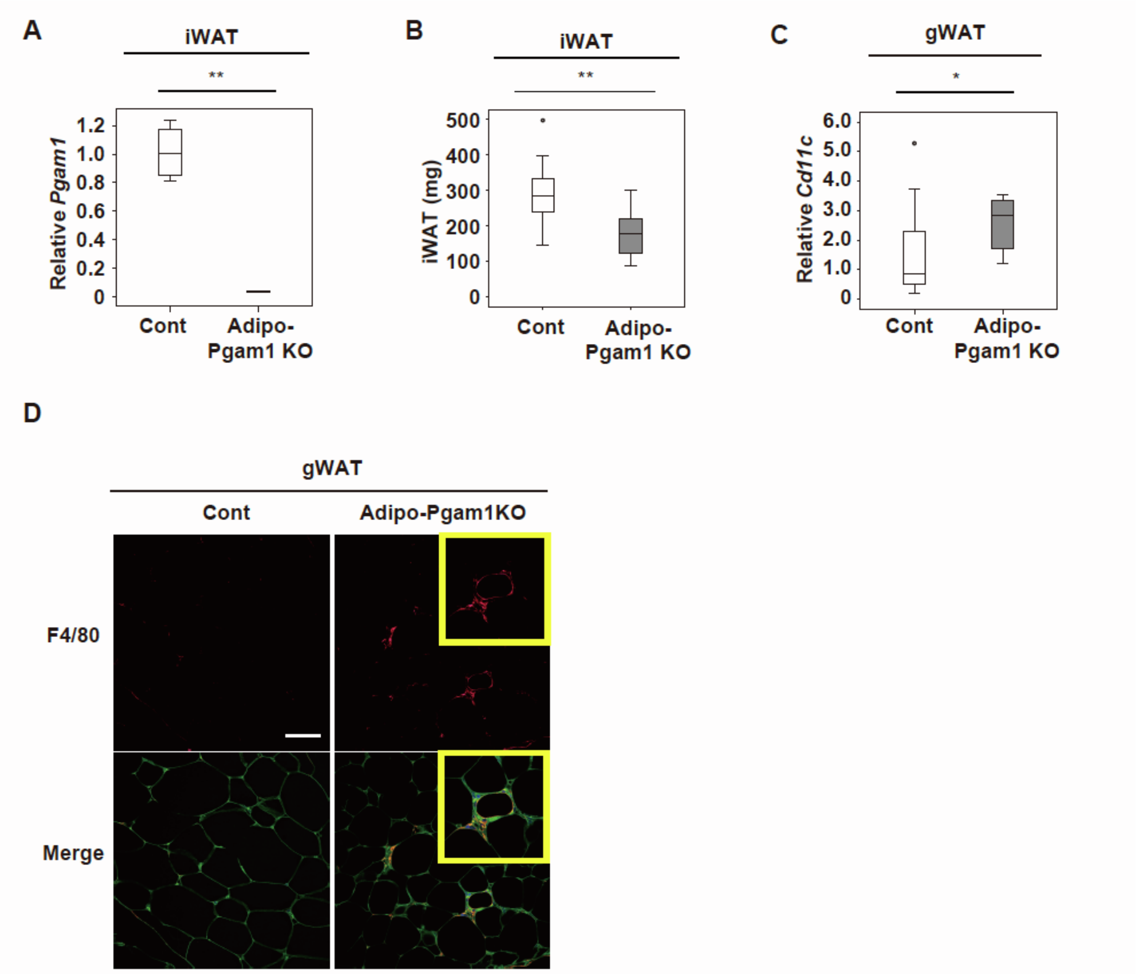

Figure S1 Adipose tissue-specific Pgam1 deletion has differing effects on BAT and WAT, Related to Figure 1

(A) Transcripts for *Pgam1* of inguinal WAT (iWAT) from Adipo-Pgam1 knockout (Adipo-Pgam1 KO) and their littermate WT (Cont) mice (n=4, 4). (B) iWAT weight of mice prepared in Supplemental Fig. 1A (n=18, 16). (C) Transcripts for *Cd11c* of gonadal WAT (gWAT) from Adipo-Pgam1 knockout (Adipo-Pgam1 KO) and their littermate WT (Cont) mice (n=14, 9). (D) Immunostaining for F4/80 in gonadal WAT (gWAT) from Adipo-Pgam1 knockout (Adipo-Pgam1 KO) and their littermate WT (Cont) mice. Scale bar=50μm. Data were analyzed by the 2-tailed Student's t-test (A–C). *P<0.05, **P<0.01. Values represent the mean ± s.e.m. NS = not significant.

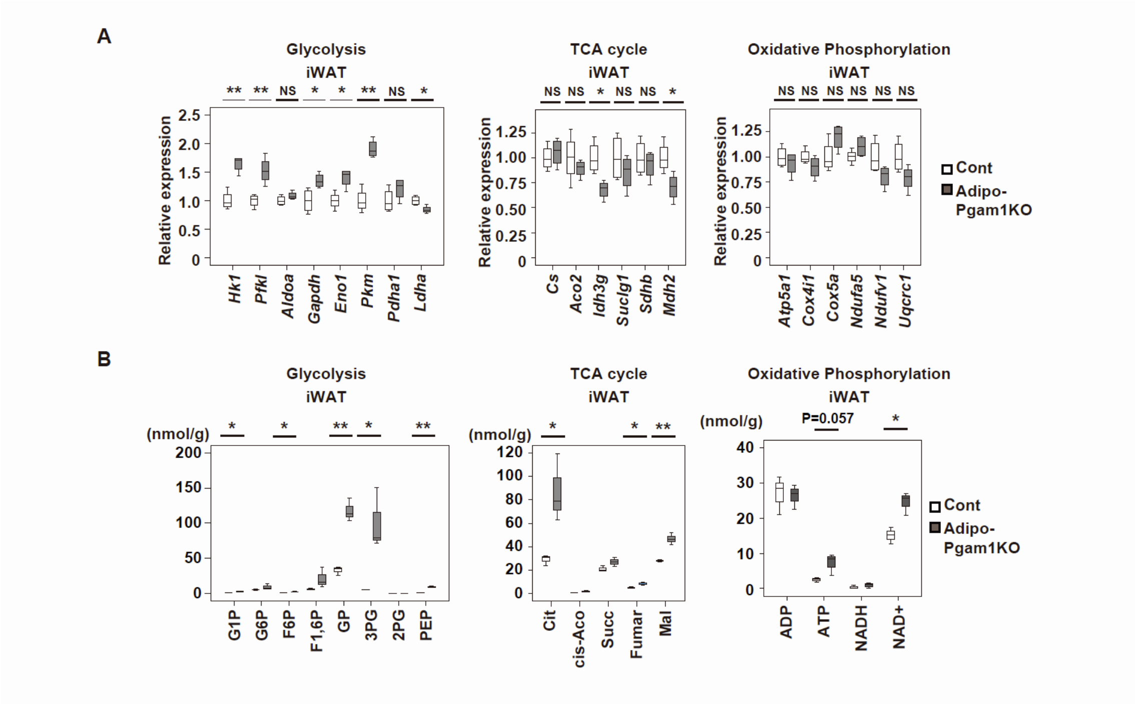

Figure S2 Opposing metabolic profiles of BAT and WAT in Adipo-Pgam1 KO mice, Related to Figure 2

(A) Transcripts for enzymes related to glycolysis, TCA cycle and oxidative phosphorylation of inguinal WAT (iWAT) from Adipo-Pgam1 knockout (Adipo-Pgam1 KO) and their littermate WT (Cont) mice (n=4,4). (B) Tissue weight-adjusted metabolite level in glycolysis, TCA cycle and oxidative phosphorylation in iWAT from mice prepared in Supplemental Fig. 2A (each n=3, 3). Data were analyzed by the 2-tailed Student's t-test (A, B). *P<0.05, **P<0.01. Values represent the mean ± s.e.m. NS = not significant.

BAT

Up-regulated genes	P-value	Q-value	Direction
Phagosome	0.000	0.000	2.898
NF-kappa B signaling pathway	0.000	0.000	3.124
Focal adhesion	0.000	0.000	2.333
Ribosome	0.000	0.000	2.547
Protein processing in endoplasmic reticulum	0.000	0.000	2.395
Regulation of actin cytoskeleton	0.000	0.000	2.127
ECM-receptor interaction	0.000	0.000	2.582
Cell adhesion molecules (CAMs)	0.000	0.000	2.086
Apoptosis	0.000	0.000	2.598
Amino sugar and nucleotide sugar metabolism	0.000	0.000	3.107
TNF signaling pathway	0.000	0.000	2.280
DNA replication	0.000	0.001	3.353
Ribosome biogenesis in eukaryotes	0.000	0.001	2.455
Cell cycle	0.000	0.001	2.085
Adherens junction	0.002	0.005	2.207
p53 signaling pathway	0.002	0.005	2.297
Sphingolipid metabolism	0.006	0.015	2.367
N-Glycan biosynthesis	0.013	0.028	2.130
Non-homologous end-joining	0.023	0.043	3.277
Other glycan degradation	0.028	0.050	2.762
One carbon pool by folate	0.034	0.058	2.616

Down-regulated genes	P-value	Q-value	Direction
Oxidative phosphorylation	0.000	0.000	3.793
Peroxisome	0.000	0.000	3.730
Valine, leucine and isoleucine degradation	0.000	0.000	3.545
Proteasome	0.000	0.000	3.414
Citrate cycle (TCA cycle)	0.000	0.000	3.879
Propanoate metabolism	0.000	0.000	3.964
Fatty acid metabolism	0.000	0.000	3.175
Pyruvate metabolism	0.000	0.000	3.142
Fatty acid degradation	0.000	0.000	2.880
Glyoxylate and dicarboxylate metabolism	0.000	0.001	3.348
Fatty acid elongation	0.000	0.002	3.414
Glycolysis / Gluconeogenesi	0.001	0.004	2.250
Sulfur relay system	0.005	0.020	4.096
SNARE interactions in vesicular transport	0.018	0.057	2.172

gWAT

Up-regulated genes	P-value	Q-value	Direction
Oxidative phosphorylation	0.000	0.000	4.766
Citrate cycle (TCA cycle)	0.000	0.000	6.724
Biosynthesis of amino acids	0.000	0.000	4.125
Ribosome	0.000	0.000	2.831
Aminoacyl-tRNA	0.000	0.000	3.530
Lysosome	0.000	0.000	2.706
Glycolysis /	0.000	0.000	3.362
Amino sugar and nucleotide sugar metabolism	0.000	0.000	3.698
Fatty acid elongation	0.000	0.001	5.085
Pentose phosphate	0.000	0.001	4.438
Purine metabolism	0.000	0.001	2.168
HIF-1 signaling pathway	0.000	0.001	2.499
Pyruvate metabolism	0.000	0.001	3.530
Fatty acid metabolism	0.000	0.001	3.328
Protein processing in endoplasmic reticulum	0.000	0.002	2.101
SNARE interactions in transport	0.002	0.011	3.362
DNA replication	0.004	0.018	3.082
Basal transcription factors	0.005	0.021	2.774
RNA degradation	0.012	0.046	2.161
Cell cycle	0.015	0.053	1.849
Apoptosis	0.016	0.053	2.029
Proteasome	0.022	0.071	2.465
Nucleotide excision repair	0.022	0.071	2.465
Ribosome biogenesis in eukaryotes	0.037	0.100	1.917
p53 signaling pathway	0.039	0.105	1.958
VEGF signaling pathway	0.047	0.118	2.034
mTOR signaling pathway	0.082	0.166	1.819

Down-regulated genes	P-value	Q-value	Direction
Valine, leucine and isoleucine degradation	0.001	0.056	3.447
Propanoate metabolism	0.009	0.164	3.373
Lysine degradation	0.030	0.313	2.343
Inositol phosphate metabolism	0.041	0.356	2.204
Ras signaling pathway	0.003	0.110	1.900
Phosphatidylinositol signaling system	0.016	0.224	2.213
FoxO signaling pathway	0.018	0.224	1.881
ABC transporters	0.019	0.224	2.598
HIF-1 signaling pathway	0.038	0.356	1.884
Adherens junction	0.003	0.110	2.624

Figure S3 Transcriptomic analysis of Adipo-Pgam1 KO mice, Related to Figure 2
The results of enrichment analysis for genes in microarray analysis in BAT (left) and gWAT (right).

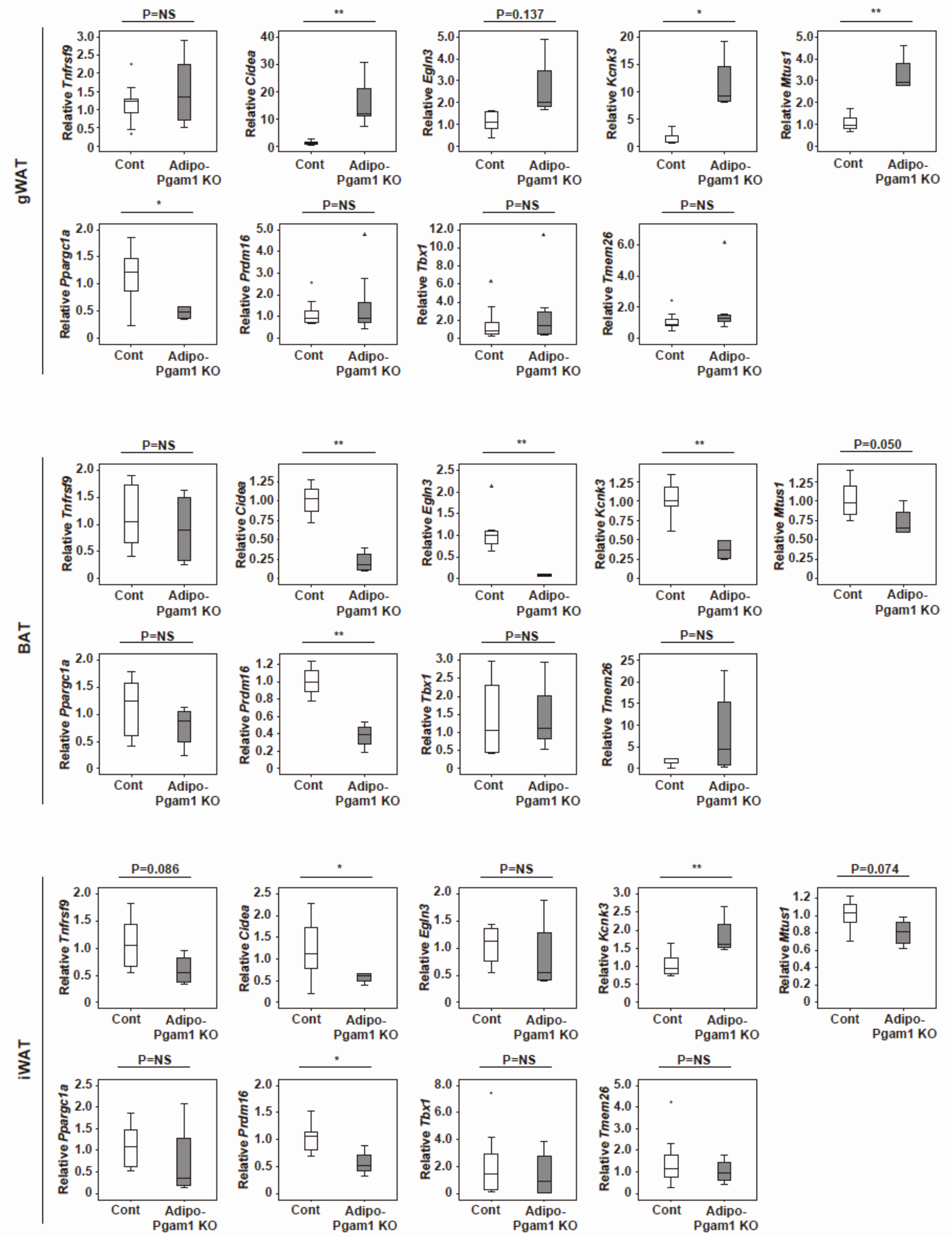

Figure S4 Expression of beige makers in Adipo-Pgam1 KO mice, Related to Figure 3

Transcripts for beige makers in gWAT, BAT, and iWAT from Adipo-Pgam1 KO mice and their littermate WT (Cont) mice (gWAT; n=14,9 for *Tnfrsf9, Cidea, Prdm16, Tbx1* and *Tmem26*, n=8,4 for *Egln3, Kcnk3, Mtus1* and *Ppargc1a*, BAT; n=8,4, iWAT; n=8,4). Data were analyzed by the 2-tailed Student's t-test. *P<0.05, **P<0.01. Values represent the mean ± s.e.m. NS = not significant.

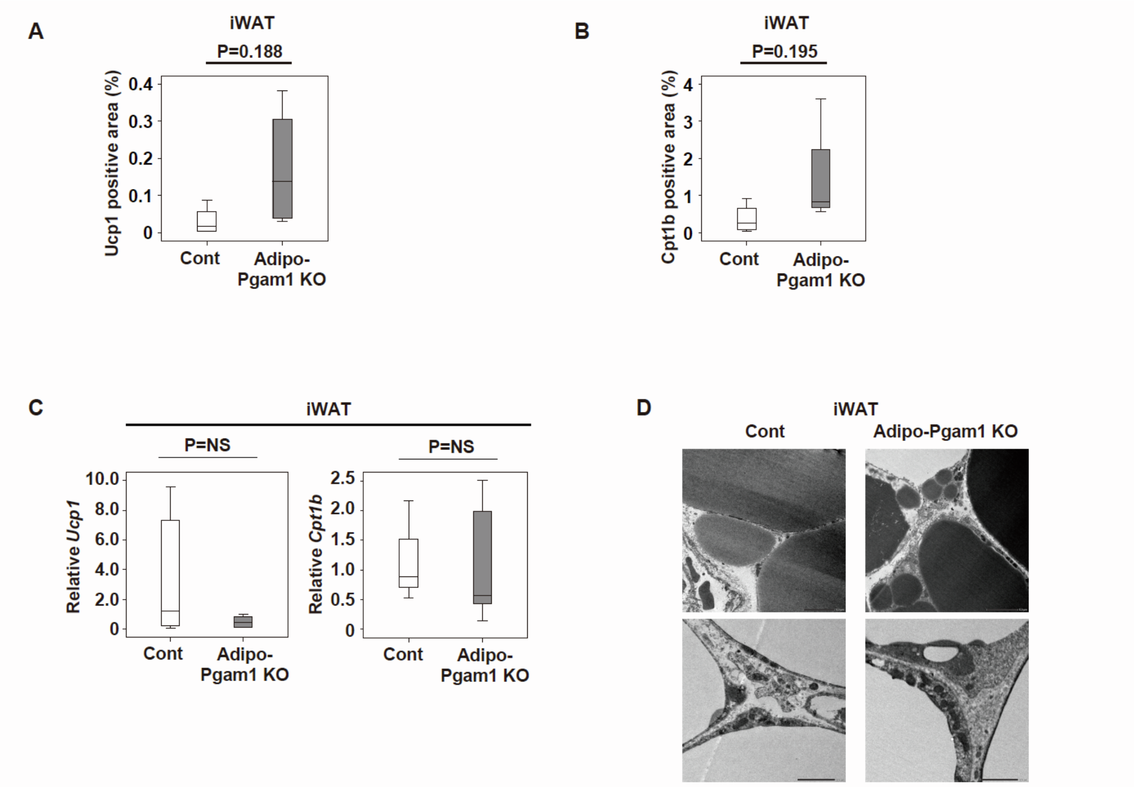

Figure S5 Metabolic profiles of iWAT in Adipo-Pgam1 KO mice, Related to Figure 3, Related to Figure 3

(A) The quantification of Ucp1 positive area (%) in immunofluorescent staining showing Ucp1 in iWAT from Adipo-Pgam1 KO and their littermate WT (Cont) mice (each n=4,4). (B) The quantification of Cpt1b positive area (%) in immunofluorescent staining showing Cpt1b in iWAT from mice prepared in Supplemental Figure 5A (each n=4,4). (C) Transcripts for *Ucp1* and *Cpt1b* of iWAT from mice prepared in Supplemental Figure 5A (*Ucp1* n=8, 4, *Cpt1b* n=3, 5). (D) Transmission electron microscopy analyzing iWAT from mice prepared in Supplemental Figure 5A. Scale bar=5µm for low magnification (upper panels) and 2µm for high magnification (lower panels). Data were analyzed by the 2-tailed Student's t-test (A–C). *P<0.05, **P<0.01. Values represent the mean ± s.e.m. NS = not significant.

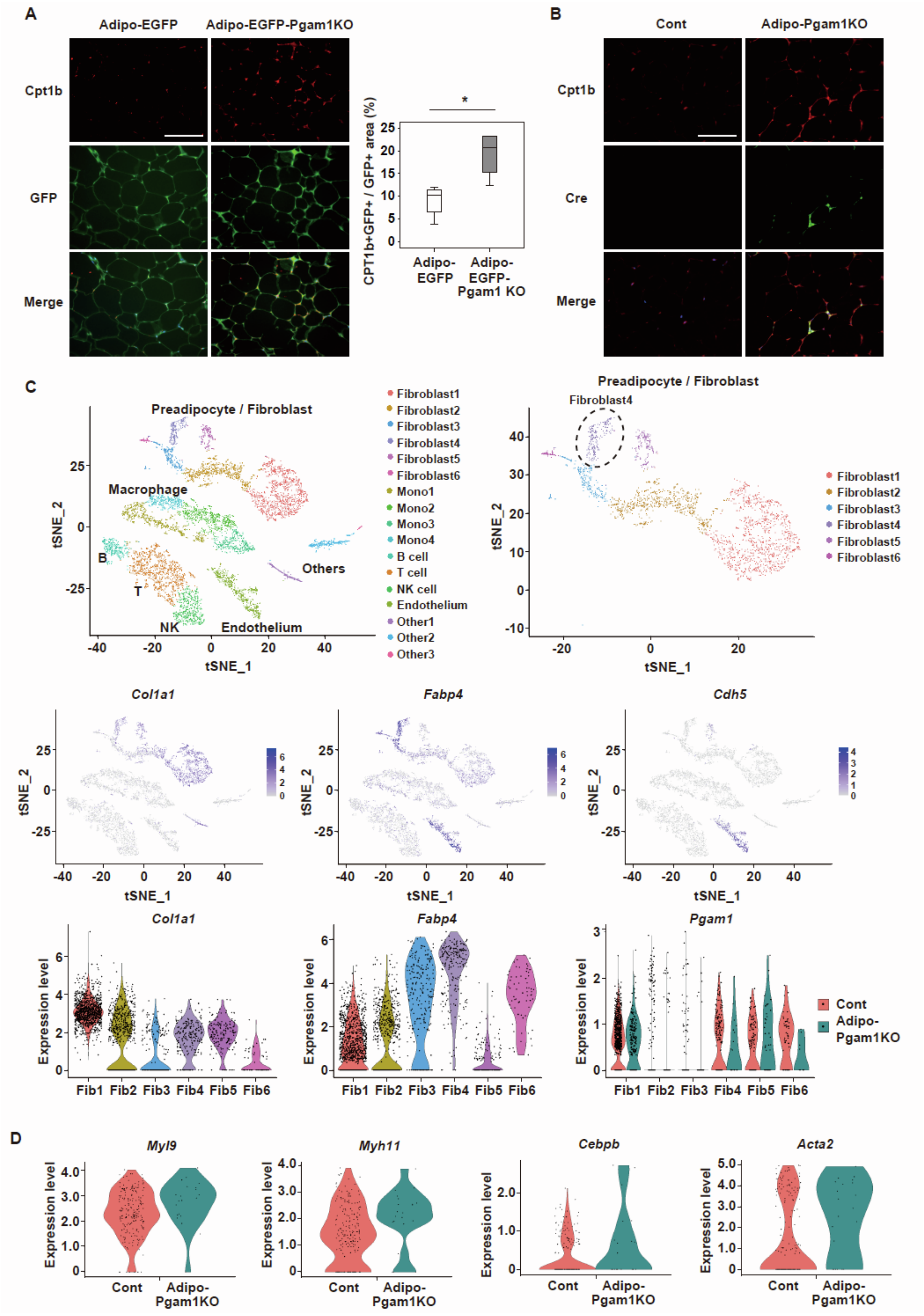

Figure S6 Pgam1 deletion induces beiging of gWAT via cell-autonomous mechanisms, Related to Figure 3

(A) Immunofluorescent staining showing Cpt1b (red) in gWAT from Adipo-EGFP and

Adipo-EGFP-Pgam1 KO mice. Scale bar=100 μm. The graphs on the right display the Cpt1b/EGFP double positive area (%) (each n=4,4). (B) Immunofluorescent staining showing Cpt1b (red) and Cre recombinase (green) in gWAT from Adipo-Pgam1 KO and their littermate control mice. Scale bar=100 μm. (C) Upper panels; Clustering of scRNA-seq analysis using gWAT from Adipo-Pgam1 KO and their littermate control mice. Middle panels; tSNE plot of scRNA-seq in gWAT from Adipo-Pgam1 KO and their littermate control mice. Lower panels; Feature plot of fibroblast and preadipocytes clusters showing *Pgam1* positive cells. (D) The violin plots showing beiging-related differential expressed genes (DEG) in the Fibroblast4 cluster. Data were analyzed by the 2-tailed Student's t-test (A, C and D). *P<0.05, **P<0.01. Values represent the mean ± s.e.m. NS = not significant.

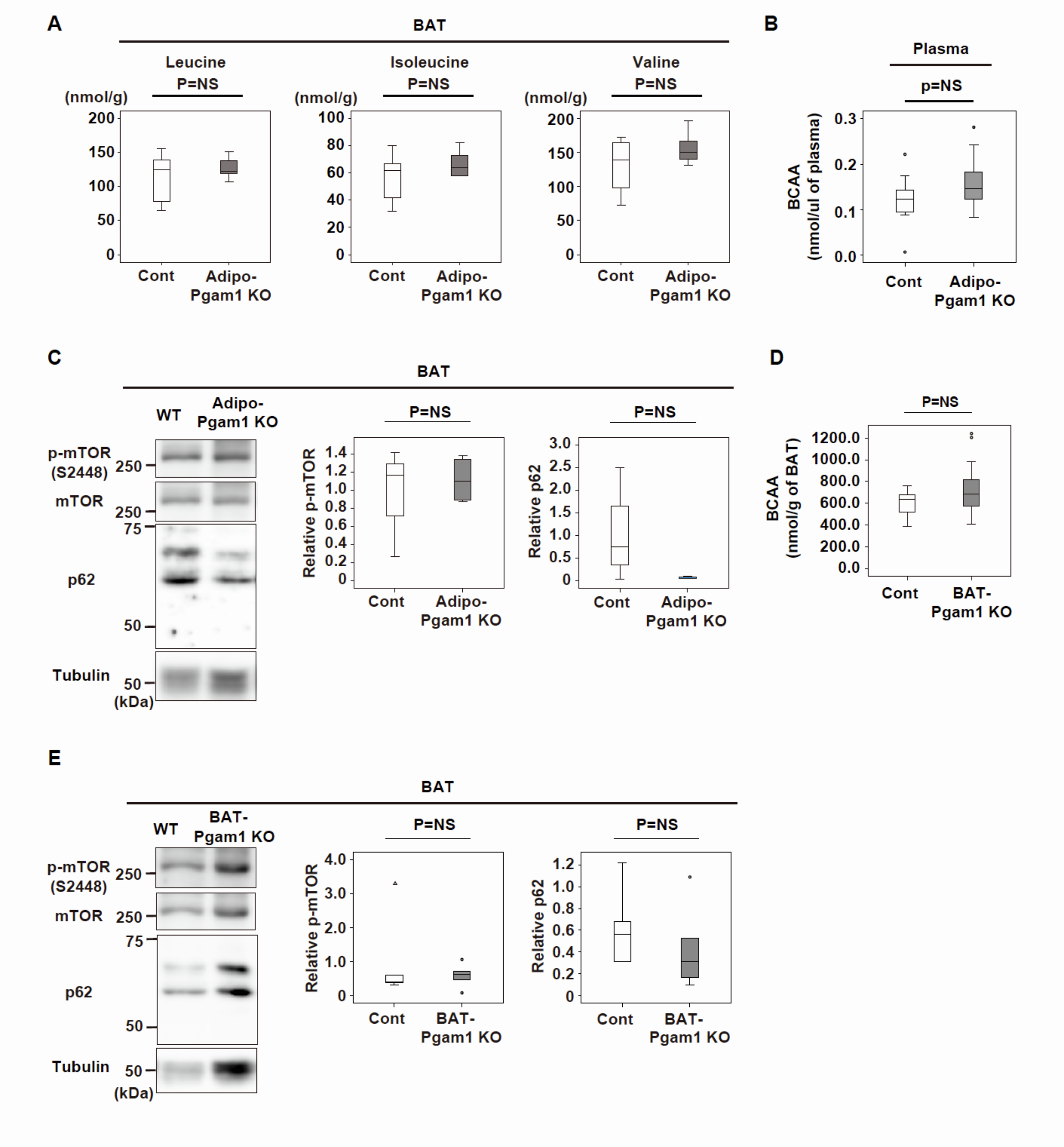

Figure S7 Effecs of Pgam1 deletion on BAT, Related to Figure 6

(A) BCAA level in BAT from Adipo-Pgam1 KO and their littermate WT (Cont) mice (n=5, 5). (B) Plasma BCAA level in Adipo-Pgam1 KO and their littermate WT (Cont) mice (n=10, 10). (C) Western blot analysis for mTOR and p62 in BAT prepared in Supplemental Figure 7A (n=4, 4). (D) BCAA level in BAT from BAT-Pgam1 KO and their littermate WT (Cont) mice (n=17, 21). (E) Western blot analysis for mTOR and p62 in BAT prepared in Supplemental Figure 7D (n=5, 5). Data were analyzed by the 2-tailed Student's t-test (A–E). *P<0.05, **P<0.01. Values represent the mean ± s.e.m. NS = not significant.

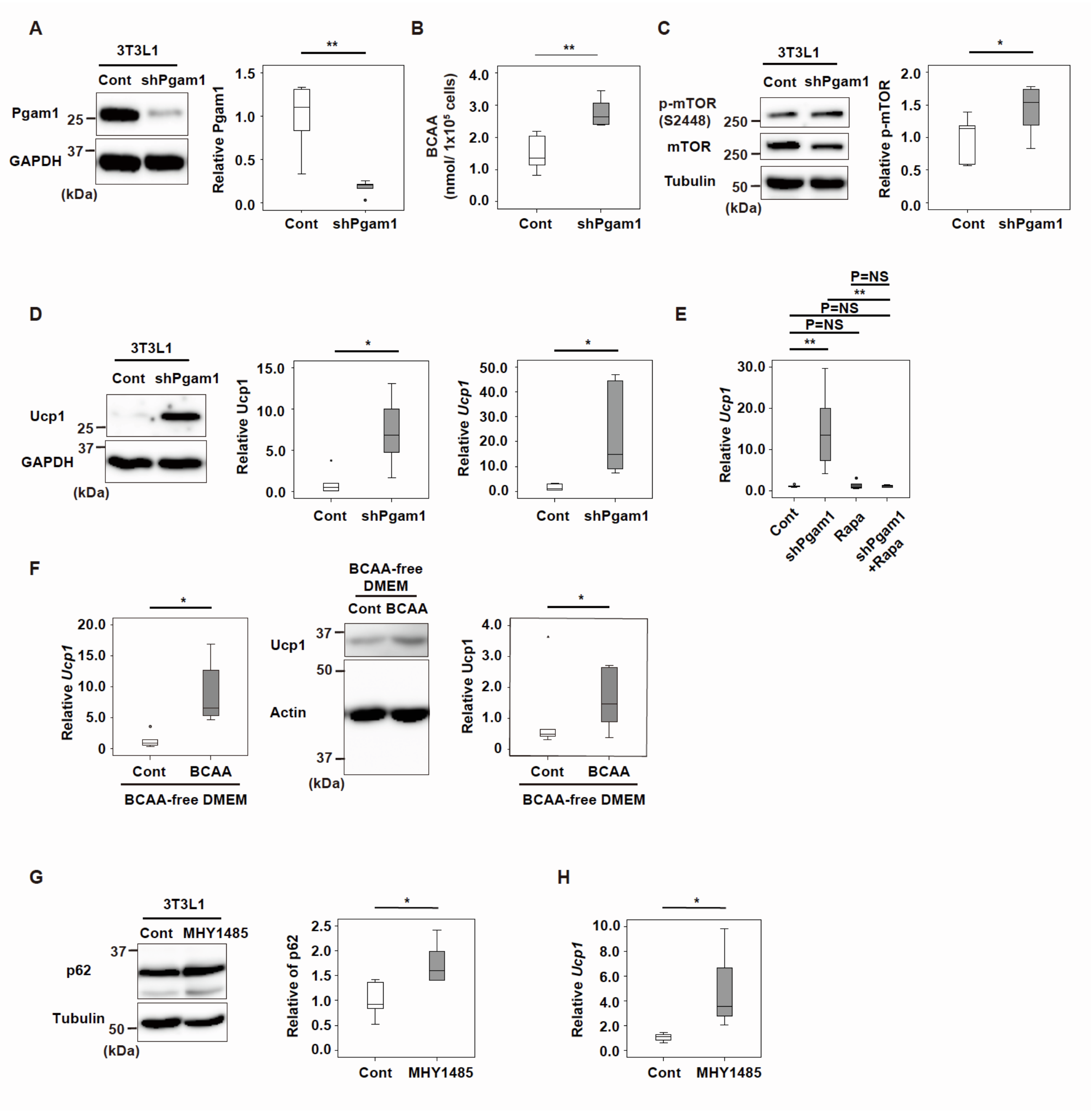

Figure S8 Effects of Pgam1 deletion on differentiated 3T3-L1 cells, Related to Figure 6

(A) Western blot analysis for Pgam1 in differentiated 3T3-L1 cells infected AAV-shPgam1 (shPgam1) or AAV-Control (Cont). GAPDH was used as loading control. Right graphs show the quantification of Pgam1 expression (n=6,6). (B) The quantification of branched amino acids (BCAA) in cells prepared in Supplemental Figure 8A (n=3,3). (C) Western blot analysis for mTOR in cells prepared in Supplemental Figure 8A. Tubulin was used as loading control. Right graphs show the quantification of p-mTOR level (n=6,6). (D) Western blot analysis for Ucp1 in cells prepared in Supplemental Figure 8A. GAPDH was used as loading control. Right graphs show the quantification of Ucp1 expression (n=6,6). (E) Transcripts for *Ucp1* of differentiated 3T3-L1 cells infected AAV-Control (Cont) or AAV-shPgam1 (shPgam1) pre-treated with or without 1 μM of Rapamycin. (n=6,6,6,6). (F) Expression of Ucp1 by differentiated 3T3-L1 cells treated with or without BCAA (the mixture of 5μM Leucine and 2.5μM Isoleucine and 5μM

Valine) (n=6,6). BCAA-free DMEM was used in this experiment. (G) Western blot analysis for p62 in differentiated 3T3-L1 cells treated with or without 2 μM of mTOR activator (MHY1485). Actin was used as loading control. Right graphs show the quantification of p62 expression (n=6,6). (H) Transcripts for *Ucp1* of cells prepared in Supplemental Figure 8G (n=3,3). Data were analyzed by the 2-tailed Student's t-test (A–H). *P<0.05, **P<0.01. Values represent the mean ± s.e.m. NS = not significant.
